# “There’s No Magic Pill to Help Me, Right?”: Sex After Early Midlife Bilateral Salpingo-Oophorectomy

**DOI:** 10.1007/s10508-026-03548-5

**Published:** 2026-10-15

**Authors:** Anuki Amarakoon, Jana Galley, Mateja Perović, Laurice Karkaby, Michelle Galper, Gillian Einstein

**Affiliations:** 1 Department of Psychology, University of Toronto, 100 St. George Street, Toronto, ON M5S 3G3 Canada; 2 Department of Psychology, University of British Columbia, Vancouver, BC Canada; 3 Rotman Research Institute, Baycrest Health Sciences, Toronto, ON Canada; 4 Tema Genus and Department of Biomedical and Clinical Sciences, Linköping University, Linköping, Sweden

**Keywords:** Risk-reducing bilateral salpingo-oophorectomy, Estradiol therapy, Sexual function, Sexual distress, DSM-5, Breast cancer mutations *(BRCA1/2)*

## Abstract

Women with risk-reducing bilateral salpingo-oophorectomy (RRBSO) report poorer sexual health than women of a similar age with intact ovaries. Since women with RRBSO lose the ability to make endogenous 17-β-estradiol (E2), open questions include whether: (1) poorer sexual function is associated with E2 loss and (2) exogenous estradiol therapy (ET_BSO_) is related to amelioration of objective decrements and subjective experiences. We combined online questionnaires with qualitative interviews to explore the sexual function of women with pathogenic variants in the *BRCA1/2* genes and RRBSO not taking ET_BSO_ (RRBSO) and those taking ET_BSO_ (RRBSO + ET_BSO_). We compared their quantitative responses to those of age-matched women with intact ovaries (AMC) and women in spontaneous menopause (SM). RRBSO had lower total sexual function scores than AMC and more sexually related distress than AMC than SM. In terms of lived experience with RRBSO + /–ET_BSO_, qualitative interviews mirrored the quantitative data with reports of pain, loss of sexual desire, and dryness, in addition to concerns about identity, relational challenges, and lacking crucial information regarding sexual response after RRBSO. The combination of the objective and subjective reports details worse sexual function and experience of younger women with RRBSO regardless of ET_BSO_. Since ET_BSO_ does not appear to ameliorate all aspects of sexual function and distress for many women with RRBSO, it is critical to consider other interventions including fully informed consent prior to RRBSO.

**Supplementary Information:**

The online version contains supplementary material available at https://doi.org/10.1007/s10508-026-03548-5.

## Introduction

Female sexual function commonly comprises six key domains: a motivation to engage in sexual activity (desire), a subjective feeling of being mentally “turned on” (arousal), a physiological genital response to arousal (lubrication), a peak sensation of extreme pleasure inducing physical and mental contentment (orgasm), a subjective evaluation of one’s sexual well-being (satisfaction), and the degree to which physiological discomfort occurs during or after vaginal penetration (pain; Meston et al., 2020). The physical discomfort of the genito-pelvic region during or after sexual activity can influence sexual satisfaction (Sorensen et al., 2018) and be part of sexual distress, the negative affective and cognitive reactions related to one’s sexual function that underlies the diagnoses of sexual dysfunction (American Psychiatric Association, 2013; Brotto, 2010; DeRogatis et al., 2002, 2008; Parish & Hahn, 2016).

Endogenous estrogens and androgens modulate sexual function in both spontaneously menopausal (SM) and surgically menopausal women (Graziottin, 2010; Kingsberg et al., 2020), with estrogens mediating vaginal congestion and lubrication and testosterone (T) promoting desire and arousal (Dennerstein et al., 1980; Nathorst-Böös et al., 1993; Semmens & Wagner, 1982; Sherwin et al., 1985). The removal of the ovaries, which produce both estrogens and androgens, can thus result in negative consequences for sexual function, including reduced sexual desire and arousal, vaginal dryness and thinning, loss of vaginal elasticity, difficulty achieving orgasm, and sexual pain (Graziottin, 2010; Simon, 2011; The North American Menopause Society, 2013). In particular, the loss of endogenous estrogens alone may contribute to dysfunction in desire, arousal, orgasm, and pain (Basson et al., 2003; Dennerstein et al., 2002; Marcinkowska et al., 2023; Modelska et al., 2004; Simon, 2011).

A notable decline in endogenous estrogens is a signal feature of all types of menopause—but not all menopauses are equivalent in their effects (Edwards et al., 2019). While SM involves a gradual reduction in endogenous estrogens and androgens during the perimenopausal period that precedes ovarian cessation, early-life bilateral salpingo-oophorectomy (BSO), the surgical removal of both ovaries and fallopian tubes prior to SM, causes an abrupt and dramatic depletion in circulating 17-β-estradiol (E2) levels—the most prominent estrogen during a women’s reproductive lifetime (Simon, 2011). Levels of E2 drop from a range of 100–150 pg/mL (at the early follicular and late follicular phases of the menstrual cycle, respectively) to less than 30 pg/mL (Graziottin & Leiblum, 2005; Simon, 2011). BSO thus results in significantly lower levels of E2 than those seen in SM (Kaur et al., 2017; Kokcu et al., 2015), where the ovaries continue to produce significant amounts of T and androstenedione, ultimately to be converted to estrogens peripherally (Fogle et al., 2007; Judd et al., 1974).

Of all the estrogens, E2 is thought to have a significant impact on postmenopausal sexual well-being. The lengthiest population-based study of the SM transition found that as women passed through the menopausal transition, they experienced significant decreases in sexual function, especially in the domains of libido and sexual responsivity, and these changes were significantly related to declining E2 levels, but not androgen levels (Dennerstein & Burger, 2001; Dennerstein et al., 2002). While androgen levels also decrease with age, they do not appear to correlate directly with sexual function (Simon, 2011). Thus, sexual function changes after early-life BSO may be more severe than that of SM women (Dennerstein et al., 2006).

Women with *BRCA1/2* mutations may elect risk-reducing BSO (RRBSO) to lower ovarian and breast cancer risk, yet they often report worse sexual health than peers with intact ovaries (Brotto et al., 2012; Finch et al., 2011; Philp et al., 2022; Stanisz et al., 2019). Referred to as “surgical menopause” when performed before SM (Kingsberg et al., 2020; National Institute for Health and Care Excellence, 2019; Edwards et al., 2019; Rocca et al., 2021), RRBSO has serious implications for women’s sexual quality of life (Elit et al., 2001; Stanisz et al., 2019). On the Sexual Activity Questionnaire (SAQ), younger women with *BRCA1/2* and RRBSO have reported declines in sexual desire, pleasure, comfort, and frequency of sexual activity a year after surgery and declines in sexual pleasure and comfort after an average of three and a half years (Finch et al., 2011; Hall et al., 2019). Compared to age-matched women (AMC) with *BRCA1/2* who retained their ovaries, RRBSO women reported more vaginal dryness and dyspareunia along with less pleasure and satisfaction during sexual activities (Madalinska et al., 2005).

In general, exogenous hormone therapy seems to improve sexual function (HT; transdermal E2, oral E2, conjugated equine estrogens; Kingsberg et al., 2020; Long et al., 2006; vaginal estrogen; The North American Menopause Society, 2022; transdermal testosterone; Parish et al., 2021, tibolone; Santoro et al., 2016). Non-randomized studies showed that RRBSO women taking different forms of exogenous estradiol therapy indicated post-RRBSO (ET_BSO_) had improved sexual function (ET_BSO_ only or combined ET_BSO_–progesterone with or without T; Finch et al., 2011; ET_BSO_ only or combined ET_BSO_–progesterone or combined ET_BSO_–T; Hall et al., 2019), reporting less bothersome sexual symptoms (systemic ET_BSO_ or topical vaginal ET_BSO;_ Tucker et al., 2016). However, others report no effect of ET_BSO_ on sexual function (combined ET_BSO_–progesterone or tibolone; Madalinska et al., 2006; ET_BSO_ type unspecified; Nathorst-Böös et al., 1993;). Using the Female Sexual Function Index (FSFI), RRBSO + /–ET_BSO_ women reported similar rates of female sexual dysfunction (Tucker et al., 2016).

Notably, ET_BSO_ might not affect sexual function domains uniformly. ET_BSO_ appears to ameliorate discomfort and dryness but not pleasure, arousal, or orgasm (Finch et al., 2011; Hall et al., 2019; ET_BSO_ only or combined ET_BSO_–progestin or tibolone; Johansen et al., 2016; Tucker et al., 2016; combined ET_BSO_–progesterone or tibolone; Vermeulen et al., 2017). Further, ET_BSO_ might not restore levels of sexual functioning to those reported presurgery (Finch et al., 2011; Hall et al., 2019; Siyam et al., 2017; Tucker et al., 2016) nor alleviate discomfort (as measured by SAQ) to the low levels observed in AMC (Johansen et al., 2016). However, one study found comparable FSFI individual domain scores between AMC and RRBSO + ET_BSO_ (ET_BSO_ only or tibolone; Islam et al., 2021).

Thus, while ET_BSO_ may alleviate pain and dryness after RRBSO, it does not ameliorate declines in desire, arousal, orgasm, or satisfaction. However, evaluation of the effectiveness of ET_BSO_ on sexual function after RRBSO is complicated by methodological differences, including the lack of distinction between various types of HT; the inclusion of women with concurrent hysterectomy; and the use of various types of scales that combine sexual function domains (e.g., SAQ merges “vaginal dryness” and “pain or discomfort during penetration” under discomfort).

Importantly, sexual distress may accompany sexual problems after RRBSO + /–ET_BSO_ (Tucker et al., 2016). When related to a loss of desire, the presence of sexual distress increases the risk of hypoactive sexual desire disorder (HSDD)—the persistent deficiency or loss of sexual thoughts, fantasies, and desire (Kingsberg et al., 2020). Compared to AMC, younger women (aged 20–49 years) with early-life bilateral oophorectomy (BO) had a higher prevalence of HSDD (Leiblum et al., 2006) that was not alleviated by ET (Dennerstein et al., 2006). However, in these studies, the absence of a reference group with intact ovaries and the inclusion of women with concomitant hysterectomy and various reasons for surgery highlights the need to further assess the role of ET_BSO_ in alleviating sexual distress after RRBSO (Dennerstein et al., 2006; Leiblum et al., 2006; Tucker et al., 2016).

Moreover, data are mixed on whether menopause caused by RRBSO confers worse sexual function or distress compared to older women in SM. Compared to SM, younger women with RRBSO and BSO for benign reasons have shown higher rates of sexual dysfunction (Alarslan et al., 2011; Benshushan et al., 2009 respectively). However, other studies have found that women with BSO for benign reasons report lower sexual desire scores than SM but better lubrication, orgasm, and intercourse frequency (Bıldırcın et al., 2020). Differences in sexual function, therefore, may vary across sexual domains and between women with RRBSO, BO, and SM. Mixed results from these studies may also be due to confounders in their BSO groups—some included women with concurrent hysterectomy; others, those who retained their fallopian tubes, a range of menopausal states prior to surgery, various reasons for surgery without specifying, various types of HT, and different ages at entering the studies.

However, it may be the case that women with early-life RRBSO, even those taking ET_BSO_, experience more sexual distress than women in SM. Women aged 50–70 with a history of early-life BO were more likely to meet criteria for HSDD than same-aged SM women (Dennerstein et al., 2006). In addition, compared to women with RRBSO performed after SM, those with early-life RRBSO were three times more likely to have sexual distress (Tucker et al., 2016). Notably, while ET was associated with lower likelihood of low desire, it did not affect the proportion of women with significant distress about their low desire, suggesting that exogenous ET_BSO_ may offset loss of function but not distress (Dennerstein et al., 2006).

Depression represents an additional factor influencing sexual function, with depressive symptoms negatively affecting RRBSO women’s sexual response (Atlantis & Sullivan, 2012; Powell et al., 2020)—an effect observed despite systemic HT_BSO_ (Chan et al., 2019). Yet, studies of sexual function or related distress after RRBSO often fail to account for depressed mood or one type of HT_BSO_ (Chan et al., 2019). This oversight is noteworthy as depressed mood increases after RRBSO (Rocca et al., 2018; Stanisz et al., 2019) with evidence of a twofold risk of new-onset depressive symptoms within 12 months post-RRBSO compared to AMC (Hickey et al., 2021b). Thus, depressed mood may contribute to sexual dysfunction and distress.

Also overlooked are how objective sexual dysfunction and distress are subjectively and relationally experienced after RRBSO. The few extant qualitative studies support some quantitative findings and thus provide a preliminary understanding of how objective changes in sexual function are experienced. Vaginal dryness is often reported, with many linking it to painful intercourse post-surgery (Hallowell et al., 2012; Hickey et al., 2021a; Meiser et al., 2000). Additionally, women report decreased sexual desire, described by some as a “complete disinterest” that consequently affects their sense of self as a woman, since desire is “the thing that makes you feel like a woman” (Hallowell et al., 2012). The loss of sexual desire may affect the dynamic of their romantic relationships, especially for those who were more sexually active before surgery; women said that their partners expected things to go back to “normal” after surgery and would “bug” them for sex (Brotto et al., 2012). Such remarks also suggest that partners lack an in-depth understanding of post-surgery changes (Brotto et al., 2012).

In general, there has been a call for greater focus on the psychosexual impact of RRBSO due to the limited qualitative research, most of which dates back more than 20 years (Brotto et al., 2012; Hickey et al., 2021a; Pernet et al., 1992). Notably, women themselves report being poorly informed and shocked by the sexual changes, saying that they faced barriers—including their embarrassment—to obtaining information and not knowing who or what to ask (Brotto et al., 2012; Hallowell, 2000).

### Current Study

Heeding this call, the primary objective of our study was to elucidate the effects of early-life RRBSO on sexual function and distress as well as its possible amelioration by exogenous ET_BSO_, by comparing scores on the FSFI and the Female Sexual Distress Scale-Revised (FSDS-R) of women with RRBSO not taking ET_BSO_ (RRBSO), those taking ET_BSO_ (RRBSO + ET_BSO_), age-matched controls (AMC), and older SM women with intact ovaries. We enhanced this analysis through the women’s own comments on their sexual function as revealed in a qualitative memory study in which they brought up their sexual function without being asked.

We hypothesized that (1) RRBSO would report lower sexual function and higher sexual distress scores than RRBSO + ET_BSO_, AMC, and SM; (2) RRBSO + ET_BSO_ would report similar sexual function and sexual distress scores as AMC; (3) RRBSO + ET_BSO_ would report higher sexual function and lower sexual distress scores than SM; and (4) women with RRBSO who spontaneously discussed their sex lives post-RRBSO would likely describe them as diminished.

Using validated measures of sexual function (FSFI) and sexual distress (FSDS-R), we compared responses of RRBSO to AMC and SM with intact ovaries who were, on average, 10 years older. Participant quotes are from semi-structured interviews that explored women's sense of their memory post-RRBSO, wherein they spontaneously discussed various aspects of sexual function (Appendix A). We compared quantitative responses with qualitative narratives to contextualize the questionnaire scores and identify any convergence or divergence of quantitative responses with qualitative experiences.

### Method

### Participants

A total of 109 participants were included in the study (103 in the quantitative component and 14 in the qualitative component). A subset of participants (*n* = 8) contributed to both components, resulting in some overlap between cohorts.

### Measures and Procedure

Participants for the online quantitative portion were recruited from Western, English-speaking countries and locally from the Greater Toronto Area (GTA) through advertorial posters around the University of Toronto campus, on the University of Toronto online bulletin, through Facebook, Instagram, and Twitter, word of mouth from friends and family, the Einstein Lab website, a Facebook *BRCA1*and *BRCA2* support group, and from our in-person cohort.

Inclusion criteria for all participants were: fluent in English and between 30 and 60 years old. Inclusion criteria for the RRBSO group were: *BRCA1/2* mutation; RRBSO before the age of 49; not less than 1-year post-surgery before entering the study; taking ET_BSO_ or not. Inclusion criteria for AMC were: regular menstrual periods and no oral contraceptives for 6 months prior to the study. Inclusion criteria for SM were: complete cessation of their menstrual cycle for at least 12 months before the study and not taking any form of HT. Screening questions to assess these criteria were part of the online questionnaire.

Participants were asked to complete the online questionnaire using Qualtrics in a quiet and private room in their own homes. Questions included the inclusion and exclusion criteria (which, if were not met, closed the survey), age, ethnicity, education, marital status, occupation, smoking behaviors, mental and physical health, and reproductive history. They completed several neuropsychological tests along with the FSFI and FSDS-R to determine sexual function and the CES-D to determine whether mood at the time of testing was associated with any results.

#### Female Sexual Function Index (FSFI)

The FSFI is a 19-item self-report inventory assessing female sexual function according to the following six domains: desire (2 items), subjective arousal (4 items), lubrication (4 items), orgasm (3 items), satisfaction (3 items), and pain (3 items). It assesses participants’ sexual function for the four weeks preceding survey completion. Using a 5-point Likert scale, participants respond to each of the items (e.g., “Over the past 4 weeks, how would you rate your level (degree) of sexual desire or interest?”); higher scores reflect higher levels of sexual function. It yields a total score (FSFI_TOTAL_) across the domains as well as individual domain scores. An FSFI_TOTAL_ score less than or equal to 26.55 is interpreted as sexual dysfunction (Meston et al., 2020). The FSFI_TOTAL_ and the six FSFI domains have demonstrated strong internal consistency (*α* ≥ .82; Rosen et al., 2000).

#### Female Sexual Distress Scale-Revised (FSDS-R)

The FSDS-R assesses personal distress related to sex; it has 13 items quantifying the frequency of negative emotions or sexual problems over the past month (e.g., “In the past 30 days how often did you feel worried about sex?”; Carpenter et al., 2015; DeRogatis et al., 2011). Items are scored on a 5-point rating scale (0 = Never, 4 = Always). Total scores range from 0 to 52, with higher scores indicating greater sexual distress and scores ≥ 11 indicating risk of clinically significant sexual distress and sexual dysfunction **(**DeRogatis et al., 2008). The FSDS-R has demonstrated strong internal consistency (*α* > .86; DeRogatis et al., 2008).

#### Center for Epidemiologic Studies Depression Scale (CES-D)

The CES-D is a 20-item self-report inventory used to measure mood by evaluating the frequency of events and ideas over the previous week (Jiang et al., 2019; Knight et al., 1997). Each of the 20 items (e.g., “during the past week, I feel that I could not shake off the blues even with help from my family or friends”) is assessed using a 4-point Likert scale (0 = rarely or none of the time, 3 = most or all of the time). Total scores range from 0 to 60, with a higher score indicating greater depressive mood (Knight et al., 1997); scores equal to or higher than 16 indicate a risk of clinical depression (Radloff, 1977; Roberts et al., 1983). The CES-D has demonstrated strong internal consistency in community and clinical samples (*α* = .85–.90; Lewinsohn et al., 1997; Radloff, 1977).

### Statistical Analysis

Cohort demographic variables between groups (RRBSO, RRBSO + ET_BSO_, AMC, and SM) were assessed by analysis of variance (ANOVA) for continuous variables and Fisher’s exact tests for categorical variables. Bivariate correlations among age, CES-D, the FSFI_TOTAL_, the six FSFI domains (desire, arousal, lubrication, orgasm, satisfaction, and pain), and the FSDS-R across the entire sample were assessed using Pearson’s *r* coefficients.

General linear models were conducted to examine the relationship between group membership and sexual outcomes, as measured by the FSFI total (FSFI_TOTAL_) and FSFI domains (desire, arousal, lubrication, orgasm, satisfaction, and pain), and the revised FSDS-R, while controlling for age and depressive symptoms (CES-D). Post hoc pairwise comparisons were made across all linear models using the emmeans package (Lenth & Piaskowski, 2025), with *p*-values adjusted for multiple comparisons using the Holm–Bonferroni correction. Internal consistencies were assessed by Cronbach’s coefficient alpha (*α*). Statistical significance was defined as *p* < .05. A sensitivity power analysis conducted in G*Power indicated that, with *α* = .05 and 80% power, the study could detect a minimum omnibus group effect of *f* = .34 for FSFI_TOTAL_ (*n* = 98) and *f* = .33 for FSDS (*n* = 103). Thus, the study was adequately powered to detect effects of moderate to large magnitude. R 4.2.0 was used for all statistical analyses.

### Qualitative Analysis

The goal of qualitative research is to describe and understand the phenomenon under study (Braun & Clarke, 2006, 2019; Downe-Wamboldt, 1992). Our phenomenon was the embodied aspects of memory post-RRBSO. Our qualitative method was informed by phenomenological inquiry, aiming to understand the lived and corporeal experience of phenomena (Merleau-Ponty, 1962; Wojnar & Swanson, 2007). As is well documented in qualitative research, other topics than the particular phenomenon under study can arise during interviews reflecting what is uniquely important to participants (Hutchinson & Wilson, 1992; Karnieli-Millter et al., 2009; Sheppard & Raby, 2024). Qualitative data in this paper reflect this; while we did not have any prompts or questions about sexual function, many women brought this up spontaneously, reflecting the importance of this aspect of post-RRBSO embodiment to them.

Participants were drawn from a larger cohort of women with *BRCA1/2* with and without RRBSO who were originally recruited from the GTA through the Familial Ovarian Cancer and After Care Clinic in the Department of Gynecology at Women’s College Hospital in Toronto, the Lady Douglas Hospital Research Center in Montreal, and the Genetics Program at North York General Hospital in Toronto (Gervais et al., 2020). We used purposive sampling from the larger study. Invitation to participate in the study was sent via email and included consent forms, allowing participants to examine and give consent prior to interview. Exclusion and inclusion criteria were the same as for the online study. We conducted interviews with 14 women with early RRBSO. Due to the pandemic, participants chose between Zoom or telephone for their interviews; both formats have been shown to produce reliable data (Krouwel, 2019; Novick, 2008), with phone interviews offering the added benefit of enhanced confidentiality, which can foster openness in discussing sensitive topics (Novick, 2008).

As part of the larger qualitative study, in-depth semi-structured interviews explored the sense of memory in women with early RRBSO + /–ET_BSO_ using an interview guide with prearranged yet open-ended questions to allow participants to share what they believed was most essential (Appendix A). Prompts included questions about their experience with RRBSO and any bodily and memory changes (e.g., “Have you noticed changes in your memory or concentration after surgery?”). Participants received a $20 gift card to a store of their choosing and an electronic thank-you note for their participation. Interviews were conducted until thematic saturation.

Transcripts were analyzed using thematic analysis, an iterative technique that involves methodically coding and developing themes from the interviews (Braun & Clarke, 2006). Coding was performed until coding saturation was reached, i.e., the point where no new arising themes or patterns relevant to sexual function necessitated the creation of new codes (Bowen, 2008; Grady, 1998, p. 26). Final themes were determined as a team (JG, AA, GE) to maintain consensus and consistency. Interview quotes were grammatically adjusted for clarity and adherence to their meaning.

#### Reflexivity

We actively assessed how our social position converged with the data and social world (Braun & Clarke, 2019; Macbeth, 2001). Importantly, none of us has RRBSO; one has reached menopause (GE), and the rest are premenopausal (JG, AA, LK, MP, MG). Nor do we share the firsthand experience of removing healthy tissue or, to our current knowledge, carrying the *BRCA1/2* gene variants. Interviews were conducted by a young woman (LK) who was unfamiliar with participants but whose experiences as a woman might have facilitated open dialogue. Quantitative analyses were performed by an undergraduate (AA) and psychology graduate students (MP and JG), while qualitative analyses were conducted by AA and JG, under the supervision of the Primary Investigator, a female cognitive neuroscientist (GE), ensuring data were interpreted from diverse female perspectives.

## Results

### Participant Demographics (Quantitative)

The online cohort comprised 103 participants: 17 RRBSO, 26 RRBSO + ET_BSO_, 38 AMC, and 22 SM (Tables 1 and 2). On average, participants were married, highly educated, and based on CES-D scores, were not at risk of clinical depression, except for RRBSO who were slightly above the clinical cutoff score of 16 (17.8 ± 12.50). There were no significant differences in BMI, years of education, CES-D, or marital status (all *p* > .05; Tables 1 and 2). At the time of surgery, RRBSO were significantly older compared to RRBSO + ET_BSO_, *t*(39) = 2.33, *p* = .025, *d* = 0.75 (Table 1). SM were significantly older than all other groups (RRBSO: *t*(99) = 3.91, *p* < .001; *d* = 1.26; RRBSO + ET_BSO_: *t*(99) = 7.07, *p* < .001; *d* = 2.05; and AMC: *t*(99) = 6.87, *p* < .001; *d* = 1.84; Table 1).

**Table 1:** Quantitative cohort: Participant demographics

Demographic	RRBSO (*n* = 17)		RRBSO + ET_BSO_ (*n* = 26)		SM (*n* = 22)		AMC (*n* = 38)	
	*M*	(± SD)	*M*	(± SD)	*M*	(± SD)	*M*	(± SD)
Age	48.6	3.30	45.6	4.64	53.4^a^	1.40	46.4	4.24
Education	17.7	3.63	17.8	2.04	16.6	2.61	17.7	3.62
BMI	25.8	3.64	26.0	3.98	25.7	4.80	26.1	6.21
CES-D Score	17.8	12.50	12.8	8.81	15.0	12.0	12.3	9.74
Age at surgery	43.3	4.57	40.2^b^	3.75				

Education measured in years. RRBSO, risk-reducing bilateral salpingo-oophorectomy; RRBSO + ET_BSO_, women with RRBSO taking estradiol therapy (ET_BSO_); SM, spontaneous menopause; AMC, age-matched controls; M, means; SD, standard deviation

^a^SM was significantly older than RRBSO, RRBSO + ET_BSO_, and AMC (*p* ≤ .001)

^b^RRBSO was significantly older at time of surgery compared to RRBSO + ET_BSO_ (*p* = .025)

**Table 2:** Quantitative cohort: Country of residence, marital status, and cancer history

Total (*N* = 103)	RRBSO (*n* = 17)		RRBSO + ET_BSO_^c^ (*n* = 26)		SM (*n* = 22)		AMC (*n* = 38)	
	*n*	*%*	*n*	*%*	*n*	*%*	*n*	%
*Country of residence*								
Canada	16	94.12	25	96.15	20	90.91	30	78.95
Other^a^	1^b^	5.88	1	3.85	2	9.09	8	21.05
*Marital status*								
Never married	1	5.88	0	0	3	13.64	6	15.79
Separated	0	0	2	7.69	3	13.64	2	5.26
Common law	3	17.65	2	7.69	2	9.09	3	7.89
Divorced	0	0	1	3.85	1	4.55	1	2.63
Married	13	76.47	21	80.77	11	50.00	26	68.42
Widowed	0	0	0	0	2	9.09	0	0
*Cancer history*								
BC Diagnosis	5	29.41	2	7.69	0	0	0	0
BC Treatment: Surgery	2	11.76	1	3.85	0	0	0	0
BC Treatment: Surgery and chemotherapy	1	5.88	1	3.85	0	0	0	0
BC Treatment: Surgery, chemotherapy, and radiation	2	11.76	0	0	0	0	0	0
Other cancer diagnosis	2	11.76	1	3.85	2	9.09	2	5.26
Other cancer treatment: Surgery	2	11.76	1	3.85	1	4.55	2	5.26
Other cancer treatment: Surgery and radiation	0	0	0	0	1	4.55	0	0

RRBSO, risk-reducing bilateral salpingo-oophorectomy; RRBSO + ET_BSO_, women with RRBSO taking estradiol therapy (ET_BSO_); SM, spontaneous menopause; AMC, age-matched controls; BC, breast cancer

^a^Western, English-speaking country of residence outside of Canada

^b^Participant did not report their country of residence

^c^Taking concurrent androgel (*n* = 2) and tibolone (*n* = 1)

### Reliability Measures

A reliability analysis indicated excellent internal consistency for the FSFI_TOTAL_ (*α* = .97) and ranged from acceptable to excellent for the six individual domains (*α* = .78–.99) in the current cohort. The FSDS-R (*α* = .95) and CES-D (*α* = .92) also demonstrated excellent internal consistency.

### Sexual Function and Sexual Distress

Bivariate correlations among the study variables are presented in Table 3.

**Table 3:** Mean (± SD) scores on the FSFI, FSDS-R, and CES-D by group

Scale	RRBSO (*n* = 17)		RRBSO + ET_BSO_ (*n* = 26)		SM (*n* = 22)		AMC (*n* = 38)	
	*M*	(± SD)	*M*	(± SD)	*M*	(± SD)	*M*	(± SD)
FSFI Total^a^	11.5^*^	8.24	17.9^*^	10.21	18.1^*^	9.23	20.9^*^	9.66
FSFI Desire^b^	1.9	1.06	2.5	1.25	2.4	0.86	2.8	1.00
FSFI Arousal^c^	1.7	1.52	2.8	1.86	2.8	1.85	3.2	1.8
FSFI Lubrication^d^	1.9	2.10	3.3	2.19	3.3	2.24	4.0	2.26
FSFI Orgasm^e^	2.2	2.18	3.3	2.15	3.3	2.21	3.7	2.1
FSFI Satisfaction^f^	2.4	1.48	3.1	1.60	3.0	1.53	3.9	1.51
FSFI Pain^g^	1.5	1.90	2.9	2.76	3.2	2.77	3.8	2.63
FSDS-R	25.0^*^	12.17	15.9^*^	9.54	13.6^*^	9.31	10.0	8.39
CES-D	17.8	12.50	12.8	8.81	15.0	12.0	12.3	9.74
Age	48.6	3.30	45.6	4.64	53.4	1.40	46.4	4.24

FSFI, Female Sexual Function Index, including Total score and six domain scores: Desire, Arousal, Lubrication, Orgasm, Satisfaction and Pain (higher scores indicate better sexual function); FSDS-R, Female Sexual Distress Scale-Revised (higher scores indicate greater sexual distress); CES-D, Center for Epidemiologic Studies Depression Scale (higher scores indicate greater depressive mood); RRBSO, risk-reducing bilateral salpingo-oophorectomy; RRBSO + ET_BSO_, women with RRBSO taking estradiol therapy; SM, spontaneous menopause; AMC, age-matched controls. Participants with missing data for the study variables were excluded from each model using listwise deletion: FSFI_TOTAL_ *n* = 98; FSFI desire *n* = 102; FSFI arousal *n* = 101; FSFI lubrication *n* = 101; FSFI orgasm *n* = 101; FSFI satisfaction *n* = 98; FSFI pain *n* = 100

^a^Denotes missing data on the FSFI_TOTAL_ (*n* = 4 AMC and *n* = 1 SM)

^b^Denotes missing data on the FSFI desire (*n* = 1 AMC)

^c^Denotes missing data on the FSFI arousal (*n* = 1 AMC and *n* = 1 SM)

^d^Denotes missing data on the FSFI lubrication (*n* = 1 AMC and *n* = 1 SM)

^e^Denotes missing data on the FSFI orgasm (*n* = 1 AMC and *n* = 1 SM)

^f^Denotes missing data on the FSFI satisfaction (*n* = 4 AMC and *n* = 1 SM)

^g^Denotes missing data on the FSFI pain (*n* = 2 AMC and *n* = 1 SM)

^*^Denotes scores indicative of sexual dysfunction (FSFI_TOTAL_ ≤ 26.55; FSDS-R scores ≥ 11)

### Female Sexual Function Index

**Fig. 1:**
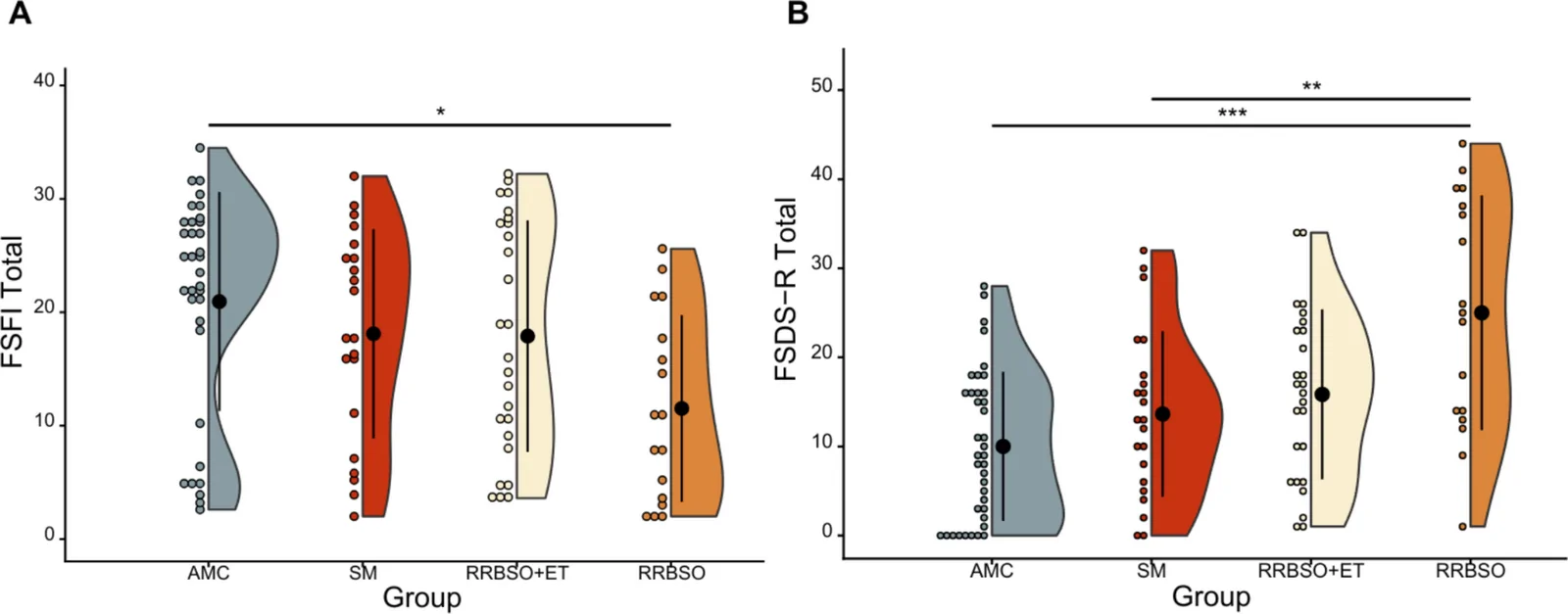
FSFI Total and FSDS-R Scores. *Note.* This figure demonstrates *g*roup differences in sexual function and distress. RRBSO had significantly lower scores than AMC on the Female Sexual Function Index (FSFI) Total (**A**) and significantly higher scores than AMC and SM on the revised Female Sexual Distress Scale (FSDS-R) **B**. Small circles represent individual data points, and violin plots depict the distribution of scores for each group. Black dots and vertical lines indicate group means ± 1 SD. Horizontal lines indicate significant pairwise group differences after Holm–Bonferroni correction. AMC, age-matched controls; SM, spontaneous menopause; RRBSO + ET, women with risk-reducing bilateral salpingo-oophorectomy taking estradiol therapy (ET_BSO_); RRBSO, women with risk-reducing bilateral salpingo-oophorectomy. * adjusted *p* < .05; ** adjusted *p* < .01; *** adjusted *p* < .001

Participants in the online cohort with missing data for the study variables were excluded from each model using listwise deletion (Table 4). All groups had average FSFI_TOTAL_ scores indicative of sexual dysfunction (≤ 26.55; Table 4). Sexual dysfunction outcomes were predicted by group while controlling for age and CES-D scores using general linear models. There was a significant main effect of group on FSFI_TOTAL_ scores after adjusting for age and CES-D, *F*(3, 92) = 2.91, *p* = .039, partial *η*^2^ = .09, indicating a moderate effect. After multiple comparison corrections, group differences emerged between RRBSO and AMC on the FSFI_TOTAL_, where RRBSO had significantly lower FSFI_TOTAL_ scores than AMC (adjusted mean difference = − 7.75, *SE* = 2.81, *t*(92) = − 2.76, adjusted *p* = .042), with a large effect size, *d* = − 0.84, 95% CI [− 1.47, − 0.22] (Fig. 1A). Non-Holm–Bonferroni corrected results are reported in Supplementary Materials.

**Table 4:** Bivariate correlations among continuous variables

Variable	1	2	3	4	5	6	7	8	9	10
Age	–									
CES−D	0.01	–								
FSFI total	−0.09	−0.31^**^								
FSFI desire	−0.18	−0.16	0.70^***^	–						
FSFI arousal	−0.07	−0.26^**^	0.93^***^	0.65^***^	–					
FSFI lubrication	−0.04	−0.28^**^	0.93^***^	0.57^***^	0.86^***^	–				
FSFI orgasm	−0.05	−0.22^*^	0.86^***^	0.51^***^	0.86^***^	0.83^***^	–			
FSFI satisfaction	−0.18	−0.38^***^	0.73^***^	0.48^***^	0.60^***^	0.58^***^	0.48^***^	–		
FSFI pain	−0.11	−0.27^**^	0.86^***^	0.52^***^	0.69^***^	0.74^***^	0.57^***^	0.63^***^	–	
FSDS−R	0.07	0.45^***^	−0.36^***^	−0.31^**^	−0.30^**^	−0.30^**^	−0.25^*^	−0.44^***^	−0.30^**^	–

CES-D, Center for Epidemiologic Studies Depression Scale; FSFI, Female Sexual Function Index; FSDS-R, Female Sexual Distress Scale-Revised. Bivariate correlations among continuous study variables are presented for the full sample (*n* = 103) ^*^p < .05; ^**^p < .01; ^***^p < .001

**Fig. 2:**
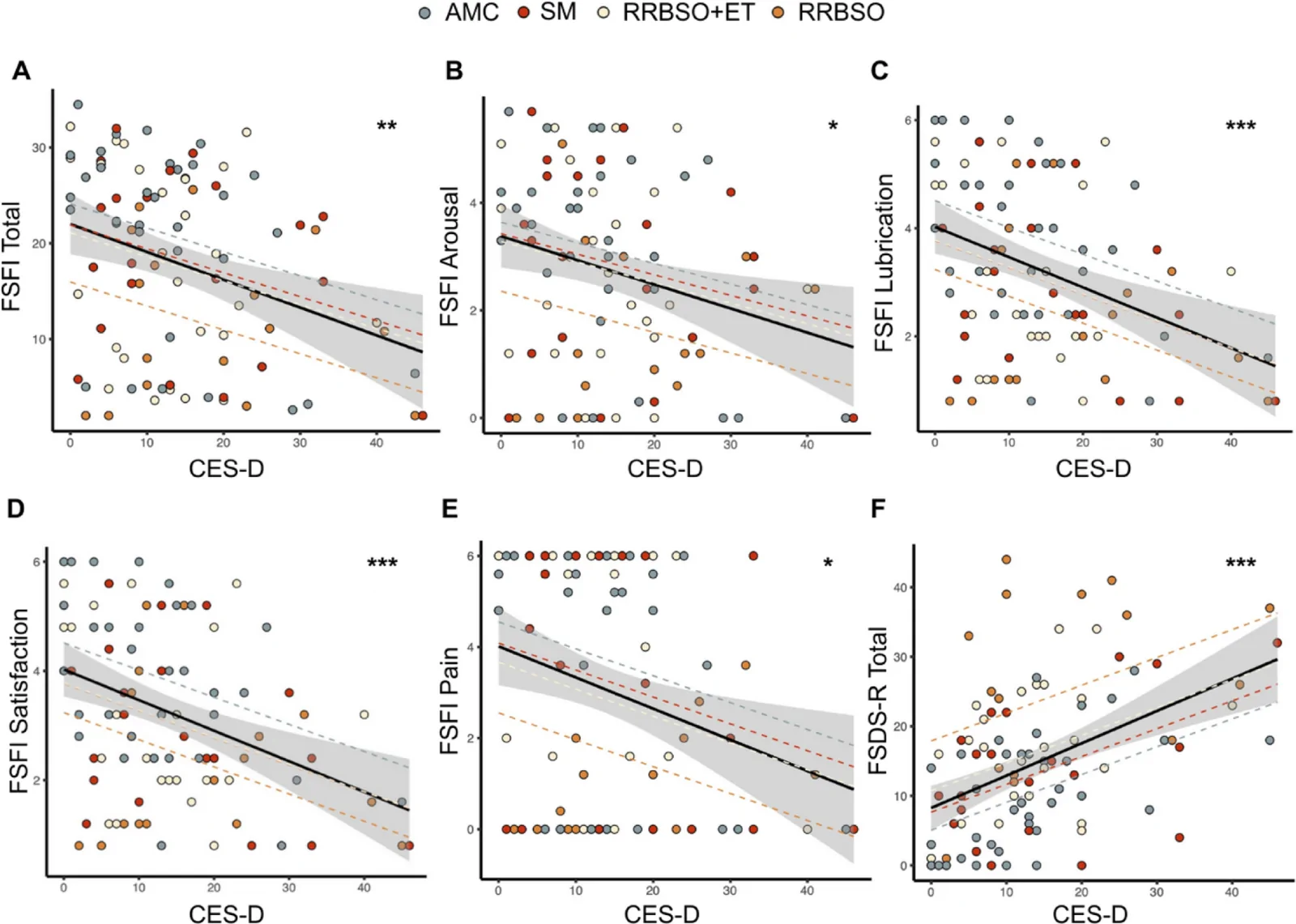
Relationship between CES-D and FSFI and FSDS-R.* Note.* This figure demonstrates the relationship between depressive symptoms and sexual function and distress. Depressive symptoms, as measured by the Center for Epidemiologic Studies Depression Scale (CES-D), are negatively associated with Female Sexual Function Index (FSFI) Total, (**A**), FSFI arousal, (**B**), FSFI lubrication (**C**), FSFI satisfaction (**D**), FSFI pain (**E**), and positively associated with the revised Female Sexual Distress Scale (FSDS-R) scores (**F**). Thick lines represent marginal model estimates; shaded regions represent 95% confidence intervals. AMC, age-matched controls; SM, spontaneous menopause; RRBSO + ET, women with risk-reducing bilateral salpingo-oophorectomy taking estradiol therapy (ET_BSO_); RRBSO, women with risk-reducing bilateral salpingo-oophorectomy. * *p* < .05; ** *p* < .01; *** *p* < .001

Additionally, there were significant main effects of CES-D on most outcomes of interest (Fig. 2). As depressive symptoms increased, FSFI_TOTAL_ (*B* = − 0.25, SE = 0.09, *t*(92) = − 2.87, *p* = .005, 95% CI [− 0.43, − 0.08]; Fig. 2A), FSFI arousal (*B* = − 0.04, SE = 0.02, *t*(95) = − 2.31, *p* = .023, 95% CI [− 0.07, − 0.01] Fig. 2B), FSFI lubrication (*B* = − 0.05, SE = 0.02, *t*(95) = − 2.46, *p* = .016, 95% CI [− 0.09, − 0.01] Fig. 2C), FSFI satisfaction (*B* = − 0.05, SE = 0.01,* t*(92) = − 3.70, *p* < .001, 95% CI [− 0.08, − 0.02] Fig. 2D), and FSFI pain (*B* = − 0.06, SE = 0.02,* t*(94) = − 2.50, *p* = .014, 95% CI [− 0.11, − 0.01] Fig. 2E) scores decreased (indicating greater sexual pain). CES-D had no significant effect on FSFI desire or FSFI orgasm (all *p* > .0.5). There were no significant associations between FSFI_TOTAL_ or any FSFI domains and age (all *p* > .05).

### Female Sexual Distress Scale-Revised

All participants in the online cohort completed the FSDS-R (*n* = 103; Table 4). RRBSO, RRBSO + ET_BSO_, and SM had average FSDS-R scores suggestive of clinically significant sexual distress (≥ 11; DeRogatis et al., 2008; Table 4). FSDS-R was predicted by group while controlling for age and CES-D scores using general linear models. There was a significant main effect of group on FSDS scores after adjusting for age and CES-D, *F*(3, 97) = 8.49, *p* < .001, partial *η*^2^ = .21, indicating a large effect. After multiple comparison corrections, RRBSO had significantly higher FSDS-R scores than AMC (adjusted mean difference = 12.35, SE = 2.69, *t*(97) = 4.59, adjusted *p* < .001, *d* = 1.39, 95% CI [0.76, 2.02]) and SM (adjusted mean difference = 11.22, SE = 3.09, *t*(97) = 3.64, adjusted *p* = .002, *d* = 1.26, 95% CI [0.55, 1.98]; Fig. 1B). There were no other significant group differences (all adjusted *p* > .05). Non-Holm–Bonferroni corrected results are reported in Supplementary Materials.

Additionally, CES-D scores were a significant covariate, *F*(1, 97) = 22.75, *p* < .001, partial *η*^2^ = .19, indicating a large effect. As depressive symptoms increased, FSDS-R scores increased (*B* = 0.40, SE = 0.08, *t*(97) = 4.77,* p* < .001, 95% CI [0.24, 0.57]; Fig. 2F). Age was not significantly associated with the FSDS-R (*p* = .378).

#### Participant Demographics (Qualitative)

Fourteen women participated in interviews; they were on average 42.43 (SD = 3.55) years old, with RRBSO at an average age of 37.93 (SD = 3.22), and the majority (85.7%) were taking ET_BSO_ (Table 5). It is also worth noting that the group of interviewees were significantly younger at age of surgery than the quantitative RRBSO group (*t*(99) = 5.32, *p* < .001; *d* = 1.51). Only two had cancer previously, and all were married (Table 5).

**Table 5:** Qualitative participant demographics

Woman (*n* = 14)	ET_BSO_	Progestogen	Age_interview_	Age_RRBSO_	BMI	CES-D	Marital tatus	Cancer therapy
Amanda^a^	Yes	Yes	40	36	31.9	20.0	Married	Yes^f^
Stephanie^a^	Yes^c^	No	48	45	25.7	20.0	Married	No
Lori^a^	Yes	Yes	44	39	20.6	0.0	Married	No
Wendy^a^	Yes	No	46	39	29.9	9.0	Married	No
Miriyam^a^	Yes	No	43	40	30.5	19.0	Married	No
Rebecca^a^	No	No	46	41	29.6	8.0	Married	No
Katerina^a^	Yes	Yes^e^	42	36	22.9	0.0	Married	No
Valerie^a^	Yes	Yes	46	39	26.6	20.0	Married	No
Camille^bd^	Yes	Progestin	37	35	33.7	14.0	Married	No
Heather^b^	Yes	No	40	36	19.2	0.0	Married	No
Nadia	Yes	No	42	40	22.4	5.0	Married	No
Jessica^b^	Yes	Yes^e^	36	32	21.0	14.0	Married	No
Adrienne^h^	Yes	Yes	40	35	42.1	9.0	Married	No
Samantha^b^	No	No	44	38	22.5	13.0	Married	Yes^g^

All interviews were completed in 2021 except Camille who completed it in November 2020. ET_BSO_, estradiol therapy; RRBSO, risk-reducing bilateral salpingo-oophorectomy; BMI, body mass index; CES-D, Center for Epidemiologic Studies Depression Scale

^a^Completed online study

^b^Invited to participate in online study but did not complete it

^c^started ET_BSO_ within a week of the interview but not taking ET_BSO_ during completion of the questionnaire

^d^Taking testosterone therapy

^e^Unspecified type of Progestogen

^f^Basal cell carcinoma surgery (surgery type unspecified)

^g^Breast cancer unilateral mastectomy and radiation (1 month) and chemotherapy (5 months and 3 months)

^h^Did not spontaneously speak about her sexual life during the interview

#### Thematic Analysis

Of the 14 women interviewed, 13 brought up their sexual issues in the context of an interview about memory, underscoring the importance of changed sexual function for women with RRBSO (Table 5). From these spontaneous revelations about their sexual lives, the following six themes were developed: (1) “No appetite,” (2) “It’s dry–it’s not like it was,” (3) Sweeping pain, (4) Sagging marriage bed, (5) Sex and the self, and (6) Women care about sex.

##### Theme 1: “No Appetite”

Even when taking ET_BSO_, participants spoke about a loss of sexual desire and orgasm, with desire having vanished entirely after RRBSO and leaving in its place an emptiness where interest in sex once resided. Four participants conveyed a complete loss, using the words “no,” “none,” or “disappeared.” When elaborating, they spoke of the changes in terms of their magnitude and onset relative to their RRBSO.

Camille (ET_BSO_ + T) likened sexual desire to that of the appetite for food: “Physically, I wasn’t craving that aspect because it’s like if you were hungry or not hungry. I just felt like I had no appetite for it whatsoever.” Stephanie (ET_BSO_) similarly described a marked loss of desire saying: “I don’t have any libido. There’s nothing.” Wendy (ET_BSO_) said: “…no drive, none. You want to, but you don’t want to.” Miriyam (ET_BSO_) described it as “lack of motivation.” While Camille said it explicitly, all statements contain an underlying yearning for sexual intimacy without the desire to enact it.

Speaking to the perceived onset of the changes in sexual desire, participants said that they first experienced loss of desire after RRBSO. Camille said: “I think the sex drive piece is weird because it’s pretty immediate–like you felt one way before the surgery and almost immediately after you feel like it’s just not there at all like it was just gone.” She elaborated: “I think prior to having the oophorectomy it wasn’t like I had a crazy high sex drive to begin with, but it was almost like what I did have just completely disappeared."

About her T therapy, Camille expressed ambivalence when she described it as an “artificial trigger” for sex. When describing how it feels to rely on T to induce sexual drive, she said, “To not have it naturally occurring you’re like mixed up in your feelings. Is this a commentary on how I truly feel or is this just literally physiological and it’s resulting in these kinds of numb feelings?” Camille described that despite T improving her desire, sex continues to lack the affective and sensory components. Thus, despite feeling desire, her sexual experience echoed the lack of sensation and fullness experienced by others and her past self.

##### Theme 2: “It Was Dry–It’s Not Like It Was”

Participants also mentioned having vaginal dryness. It was clear to them that the dryness, like loss of desire, was linked to RRBSO. Wendy (ET_BSO_) said she had “vaginal dryness and all that kind of stuff.” Nadia (ET_BSO_) similarly described that she has “to be careful with hygiene in terms of vaginal dryness and itching.”

While some RRBSO + ET_BSO_ still experienced vaginal dryness, others did not. Katerina (ET_BSO_) reflected on what she heard in the media about menopause and remembered “commercials of vaginal dryness and sexuality” which shaped her expectation of vaginal dryness following her surgery. Yet, her own experience contradicted her expectation: “I would say [vaginal dryness is] what pops up the most. And then having been through it myself, it was the complete opposite. So, I had none of those symptoms. I’m not getting the dryness; it hasn’t affected me.”

Camille said: “I know things like dryness can be an issue or pain during sex or that sort of thing in terms of that, I feel lucky, I feel good. I don’t have the vaginal dryness.” Thus, these narratives suggested that ET_BSO_ may be effective in preventing vaginal dryness in some women but for others like Wendy, Nadia, and Miriyam, they experienced dryness despite taking ET_BSO_.

##### Theme 3: Sweeping Pain

Virtually everyone described sexual pain, to which they looked for solutions. They spontaneously discussed how they experienced pain within the body, along with its impact and role within their lives. Stephanie (ET_BSO_) said, “I have burning. Like vaginal burning.” Nadia (ET_BSO_) said, “If I don’t have intercourse for like a month that’s going to be a lot more painful than if I didn’t in my twenties.” Pain during intercourse may be routine enough that it was anticipated and met with potential solutions.

However, while most participants described very similar experiences of burning or sexual pain, they understood, conceptualized, and ultimately used it differently to inform their course of action. Instead of allowing the anticipation of pain to serve as a deterrent to sexual activity, Nadia used it a motivator for maintaining regular sexual activity. In reference to the fact that sex is more painful after a period of celibacy, she stated, “I’m trying to stay sexually active. Trying to, to keep things in check that way. I definitely have to keep in mind to be sexually active.”

Samatha’s (no ET_BSO_) pain during sexual activity drove her to go to a menopause clinic: “I remember being referred to a menopause clinic and it was specifically because it was excruciatingly painful for me to have sex. And I was like “what am I going to do?” Like this [is] awful.” She stated: “Sex is extremely painful for me, so I have to work really hard at different options of trying to alleviate some of that.” The words “I have to work” convey that Samantha deemed the pain experienced during sex as something over which she had the power and ability to ameliorate. Even so, she did not describe the solution as easy, saying the alleviation necessitated “really hard” work.

Wendy’s (ET_BSO_) pain was positioned within the context of her RRBSO, contoured by the reminder of her risk of cancer. Wendy said the loss of sex drive “can be painful,” associating its loss to the sociosomatic aspects of pain. She added: “I think the mental aspect gets into it a lot too because then you remember oh why, why, why does it hurt? Oh, that’s why.” For Wendy, the weight of cancer risk was not removed along with her ovaries—its psychological grip followed her into the bedroom, compromising her sexual function.

##### Theme 4: Sagging Marriage Bed

Women mentioned maneuvering RRBSO-associated changes within the context of their marriage. Nadia (ET_BSO_) urged women to “keep in mind whether your relationship is a great supportive resilient one or one that’s really highly dependent on your physical appearance or the amount of sex that you can have.” Similarly, Stephanie (ET_BSO_) warned that for “…women that maybe don’t have good relationship with their husband, [it] can go downhill. My husband and I have been through a lot of relationship struggles in this.” Both accounts emphasized the role sexual struggles play in their marital relationship following RRBSO. A nurturing and adaptable relationship going into the surgery may serve as a support during struggles with sexual response. Yet, for any marital relationship, RRBSO and associated sexual difficulties can catalyze more obstacles to intimacy and exacerbate sexual difficulties.

Amanda (ET_BSO_) said:

My husband was nothing but supportive and encouraged [me] to do the surgery right away. So, there was no hesitation even on his part. But you know, I’m sure that had an effect on him as well right? You can deal with [hot flashes] in that they don’t cause an effect on anybody else right? Whereas you know, obviously, vaginally I feel like it might have had an effect on my marriage.

Rebecca (no ET_BSO_) was “surprised” by the loss of physical intimacy with her husband: “I’m not talking about only sexual intimacy. Like no, like barely any touching at all… that’s the thing more that surprised me.” Like Amanda, she also suspected the surgery affected her husband but particularly his physical attraction toward her: “I know how I see myself, so it looks ugly, so I don’t know. Like does [my husband] also think that? I have no idea.” Thus, sexual problems reached into relational realms, affecting their partners and their relationship.

##### Theme 5: Sex and the Self

In addition to the physiological and interpersonal spheres, issues with sexual function were also situated in the sociocultural spheres of life. Nadia (ET_BSO_) elucidated how sex is a phenomenon that transcends biology, linking sexual well-being after any type of menopause with identity:

I always knew that how women were describing their experiences after they’ve had after a total hysterectomy or a total oophorectomy or after menopause that’s natural is very, very colored by what their lives have been like sexually and what their identity was, whether they thought of themselves as female or male.

Explicitly, she points to the importance of women’s ovaries for gender identity which may come into question after ovarian removal.

Wendy (ET_BSO_) integrated her dryness into a conceptualization of herself when elaborating on the physical changes in her body since RRBSO: “I guess vaginal dryness and all that kind of stuff. You just–I feel older.” Camille (ET_BSO_) explained how her mastectomy paled in comparison to thinking about “things to do with sex or sex drive or you know youth or you kind of associate that with vitality and youngness and healthy relationship sort of thing.” She further described how these conceptions of sexuality are found “in the media” and emerged in “conversations you have with your friends,” making her feel, “a little outside.” Thus, sexual activity was also aligned with younger age, as indicated by Camille’s words, “vitality” and “youngness” when describing her experience navigating sexual desire changes.

##### Theme 6: Women Care about Sex

That 13 of the 14 participants spontaneously spoke about sex when interviewed about their memory indicates that sex was important to them. In discussing sex, they mentioned knowing other women who deferred RRBSO because of fear of the ensuing sexual issues. Lori (ET_BSO_) said that she “had a friend that also had a *BRCA1/2* [mutation] and put off the surgeries […] she was worried about her sex life going downhill because that part was really important to her.” Camille (ET_BSO_) also described changes in sex drive as one of the most profound changes since RRBSO, having said, “What I feel like has the most day-to-day impact on me I would say the weight change and the sex drive. [Those] would be the two that I would say have been the highest impact.”

The discussion of sex was colored by a hunger to know and understand more about the risk to sexual function from RRBSO. Some women said that, before the surgery, they were aware of the risk to their sex lives but highlighted that there was a lack of clarity or elucidation of these risks, and a lack of conversation about female sexuality. They placed this lack of conversation at the feet of the medical community. With little to no prior knowledge of the potential changes their sex lives might suffer post-RRBSO, participants were taken aback by the silence around the sudden change to their sexual function. Miriyam (ET_BSO_) recounted her experience of seeking information from doctors and support communities regarding life after RRBSO. Valerie (ET_BSO_), despite being warned about physiological changes and menopausal symptoms such as weight management issues and hot flashes, noted that no one mentioned changes in sexual desire: “The loss of sex drive, that was a surprise. Women need to be warned that it actually could happen.”

Nadia (ET_BSO_) likened the uncertainty of sexual sequala as navigating an unknown path: “you don’t really know the road you’re going to be taking.” When asked about what felt normal since the surgery, Wendy (ET_BSO_) said, “I was told about [RRBSO]. That it can reduce [libido]. I just didn’t, really mentally understand it.” The surprise of many participants, despite some being told about it, illustrated that being aware of something and sufficiently understanding are two different things. It also showed that informed consent entails more than being told about what to expect from an intervention—rather it necessitates help in understanding the full ramifications.

Some attributed this lack of discussion to sexism. Samantha (no ET_BSO_) said:

Like a man can’t get an erection and there’s a pill out like the next week you know what I mean? But meanwhile I can’t have sex with my husband but I’m not important at all. There’s no support for me. There’s no magic pill to help me, right?

Camille yearned for a conversation about sex after RRBSO: “I would have liked to have been able to have a more open conversation I guess about the sex stuff to be quite honest.” In what appeared to be an attempt to explain its absence, she compared her conversations with women about mastectomy to those about oophorectomy, suggesting that the sexual impact of oophorectomy was too intimate:

Maybe it’s one step more personal than the breasts you know? I don’t think [sex drive] can only be related to your hormones. It has to probably be related to your mind and how you feel and other things too. So maybe that’s too personal for people, right? That’s really about your internal life not just their external body, what they’re showing to other people.

Samantha attributed this lack of discussion among women considering RRBSO to a broader lack of conversation about women’s sexual lives, revealing that even among women with intact ovaries, sexual well-being may be slighted:

I talk a lot about it in my group and on my [social media] and a lot of the experts we have in this is to talk about those things because a lot of women don’t understand when they lack a sex drive.

Camille echoed this oversight when she said, “I feel like women’s sexuality is still not a common topic for conversation. Maybe that’s why it’s avoided.” Here, Camille exposed how candid discourse and prioritization of women’s sexual lives fall short.

The narratives underscored that for these women, there was a lack of information about sexual changes and how they might actually manifest, and that sex carried weight and meaning beyond the act of intercourse or sexual activity but was sewn into the fabric of marriages, relationships, and sense of self.

### Quantitative and Qualitative

#### Quantitative Scores of the Interviewees

Examining the qualitative and quantitative analysis together revealed that quantitative measures of compromised sexual function and sexual distress in women with RRBSO were supported and extended by their experience. Eight of the 14 interviewees also completed the quantitative instruments (Table 6). Six of the eight women who completed both components met the cutoff for sexual dysfunction or distress and half of these women may meet the criteria for HSDD, based on a score of ≤ 5 on the desire domain of FSFI and ≥ 11 on the FSDS-R (DeRogatis et al., 2008; Meston et al., 2020; Table 6).

**Table 6:** Integrated qualitative and quantitative results of women’s sexual response

Participant (*n* = 8)	Current HT	Qualitative	Quantitative							
			FSFI total	FSFI pain	FSFI desire	FSFI arousal	FSFI orgasm	FSFI lubrication	FSFI satisfaction	FSDS-R
Amanda	ET_BSO_ + P	“…vaginally I feel like it might have had an effect on my marriage.”	28.0	6.0	3.6	4.2	5.2	4.2	4.8	24.0*
Stephanie	ET_BSO_^a^	“I don’t have any libido. There’s nothing.”	7.7*	1.2	1.2	0.9	1.2	1.2	2.0	39.0*
Lori	ET_BSO_ + P	“I had a friend that also had a *BRCA1/2* [mutation] and put off the surgeries […] she was worried about her sex life going downhill because that part was really important to her.”	32.2	6.0	5.4	5.1	4.4	5.7	5.6	1.0
Wendy	ET_BSO_	“There’s no [sex] drive. The mental aspect gets into it a lot too because then you remember why does it hurt? Oh, that’s why.”	15.8*	0.0	1.2	3.0	5.2	3.6	2.8	24.0*
Miriyam	ET_BSO_	“…there’s a physiological change, there’s a dryness.”	18.9*	4.0	1.2	1.8	4.8	5.1	2.0	18.0*
Rebecca	No	“I’m not talking about only sexual intimacy. Like no, like barely any touching at all.”	21.4*	0.4	3.6	5.1	6.0	5.1	1.2	25.0*
Katerina	ET_BSO_ + P	“I’m not getting the dryness […] it hasn’t affected me.”	28.9	6.0	3.0	3.9	5.2	6.0	4.8	1.0
Valerie	ET_BSO_ + P	“The loss of sex drive was a surprise […] women need to be warned that it actually could happen.”	10.4*	0.0	2.4	1.5	3.6	2.1	0.8	6.0

Comparison of scores on the FSFI and FSDS-R with interview quotes for those who completed quantitative and qualitative assessments. HT, hormone therapy; ET_BSO_, estradiol therapy; P, progestogen; RRBSO, risk-reducing bilateral salpingo-oophorectomy; FSFI, Female Sexual Function Index; FSDS-R, Female Sexual Distress Scale-Revised

^a^Started ET_BSO_ within a week of the interview but was not taking ET_BSO_ during completion of the questionnaire. ^*^Met clinical cutoff score of respective quantitative instruments (FSFI_TOTAL_ or FSDS-R)

Aligned with the quantitative FSFI desire, lubrication, and pain scores, interviewees reported loss of sex drive and elevated vaginal dryness and pain even when taking ET_BSO_ at the time of their interview and when completing the questionnaire (Table 6). Two of the three women who spoke about dryness and pain also had CES-D scores ≥ 16, mirroring the finding that CES-D was associated with worse FSFI pain and lubrication scores.

While participants did not explicitly speak to their sexual satisfaction as the FSFI does, they did speak to the impact of their sexual response on their marriage and relationships, an aspect measured by the satisfaction domain of the FSFI (Meston et al., 2020). For instance, our participants suspected that the loss of physical intimacy had a negative effect on their spouse, and thus, their relationship. For some, this relational strain was distressing. Notably, those who met the FSDS-R cutoff for significant distress tended to report relational challenges.

Although participants did not explicitly mention being distressed about sex, they consistently described changes in sexual function as having a negative impact on their lives, suggestive of the presence of distress. Our participants used words like “difficult,” “awful,” and some described the change in sexual desire as having the “highest impact” post-op. For example, despite the absence of dysfunction on the FSFI_TOTAL_, Amanda had FSDS-R scores suggestive of clinically significant sexual distress and her narrative revealed how the sexual changes post-op harmed not only herself but also her husband (Table 6). Accordingly, the discrepancy between meeting the cutoff on the FSDS-R but not the FSFI_TOTAL_ (i.e., scores indicating sexual distress without sexual dysfunction) may arise from the effects of the reported sexual changes on participants' marital relationships, leading to distress without dysfunction per se.

Finally, those meeting a cutoff suggestive of sexual dysfunction on the FSFI_TOTAL_ (Valerie), FSDS-R (Amanda), or both (Stephanie, Wendy, Miriyam, Rebecca) explicitly tied their changes in sexual function to their RRBSO (Table 6). For instance, Valerie insisted that women considering RRBSO need to be made aware of the sexual sequela and Lori knew someone with BRCA1/2 who opted out of the surgery because of the fear of the sexual consequences (Table 6). Moreover, Amanda described how RRBSO affected her marriage because of its toll on her sex life, and Wendy, who had an FSFI pain score of 0, described emotional pain around her loss of sex drive and cancer risk (Table 6).

## Discussion

It has long been documented that women with risk-reducing bilateral salpingo-oophorectomy (RRBSO) have sexual problems post-surgery (Brotto et al., 2012; Finch et al., 2011; Hall et al., 2019; for RRSO see: Johansen et al., 2016; Tucker & Cohen, 2017). This study aimed to elucidate the effects of early-life RRBSO on sexual function and distress as well as its possible amelioration by exogenous ET_BSO_. To do so, we compared quantitative FSFI and FSDS-R scores across RRBSO, RRBSO taking estradiol therapy (RRBSO + ET_BSO_), AMC, and SM women, complemented by qualitative narratives in which RRBSO + /–ET_BSO_ women spontaneously described sexual issues. Consistent with the mixed literature, both the quantitative and qualitative results indicate that ET_BSO_ does not fully alleviate sexual dysfunction, suggesting that factors beyond physiological symptoms may contribute to sexually related distress; regardless of ET_BSO_, this was mirrored in the qualitative narratives in which women described their sex lives as distressing. Our novel finding that the CES-D is inversely related to the FSFI_TOTAL_, FSFI domains of sexual arousal, lubrication, satisfaction, and pain—and positively associated with the FSDS-R—suggests that treating one condition may help relieve the other. Future work should clarify the direction of this relationship. Finally, and importantly, despite previous studies documenting that women who anticipated potential sexual dysfunction after RRBSO reported no subsequent sexual distress—whereas all those who did not anticipate such changes did (Brotto et al., 2012)—our qualitative results demonstrate that women with RRBSO + /–ET_BSO_ due to *BRCA1/2* genetic mutations still feel inadequately informed about the sexual consequences of their choice for RRBSO.

### Female Sexual Function Index

The mean FSFI_TOTAL_ scores indicative of sexual dysfunction across all groups is in keeping with previous literature indicating high prevalence of sexual dysfunction among RRBSO, premenopausal, and SM women (Bober et al., 2015; Dąbrowska-Galas et al., 2019; McCool et al., 2016; Tucker et al., 2016; von Hippel et al., 2019). We hypothesized that RRBSO women would report lower sexual function than RRBSO + ET_BSO_ and AMC. Surprisingly, there was only lower sexual function in RRBSO compared to AMC; thus, ET_BSO_ may provide some relief or maintain sexual function in some women but not others.

The comparable sexual function scores observed between RRBSO + /–ET_BSO_ is consistent with previous research using the FSFI_TOTAL_ (Tucker et al., 2016). However, compared to AMC, prior studies showed worse sexual function in RRBSO women regardless of HT_BSO_ (Madalinska et al., 2005) or ET_BSO_ (ET_BSO_ alone or combined ET_BSO_ and progestin or tibolone: Johansen et al., 2016). Differences may be due to different measures and types of HT_BSO_ (SAQ; Johansen et al., 2016; HT_BSO_ unspecified; Madalinska et al., 2005). Moreover, the lack of observed difference between RRBSO + ET_BSO_ and AMC in our study may be due to greater variation in the RRBSO + ET_BSO_ group. This variation may be due to individual differences in response to ET_BSO_ and/or route of administration; topical vaginal ET_BSO_ has been linked to lower rates of sexual dysfunction compared to systemic ET_BSO_, respectively (Tucker et al., 2016). A larger cohort may be needed to detect the subtle effects of ET_BSO_.

#### FSFI Individual Domains

Consistent with previous studies, there was no significant difference in any of the FSFI individual domains between RRBSO and RRBSO + ET_BSO_ (HT_BSO_; Finch et al., 2011; Islam et al., 2021; Johansen et al., 2016; Madalinska et al., 2005; combined ET_BSO_–progesterone or tibolone; Madalinska et al., 2006; Tucker et al., 2016). However, in contrast to other studies, RRBSO + ET_BSO_ did not report better FSFI lubrication and pain scores than RRBSO (Finch et al., 2011; Hall et al., 2019; Tucker et al., 2016; Vermeulen et al., 2017). Again, these contrasting results may reflect differences in measures, as prior studies assessed pain and vaginal dryness using the SAQ, and some included various types of HT_BSO_ (Johansen et al., 2016; tibolone or ET_BSO_ and progestin; Vermeulen et al., 2017;) or ET_BSO_ (combined ET_BSO_ + T; Finch et al., 2011; Hall et al., 2019). Thus, the role of exogenous hormones on alleviating pain and improving lubrication after RRBSO remains unclear.

Contrary to our hypothesis, RRBSO did not significantly differ from AMC on any of the FSFI individual domains. These findings contrast with prior reports of reduced arousal, lubrication, orgasm, pain in RRBSO on the FSFI (Islam et al., 2021) as well as decreased pleasure and higher discomfort using the SAQ in RRBSO compared to AMC (Johansen et al., 2016). It is worthwhile noting that these two other studies also did not completely overlap in the subdomains in which they found significant differences; again, this may be due to different scales. Moreover, since neither study reported any formal adjustment for multiple comparisons, it is difficult to know whether significant results would have been maintained (Islam et al., 2021; Johansen et al., 2016).

In line with our hypothesis, however, RRBSO + ET_BSO_ and AMC did not differ significantly across the FSFI domains. Thus, ET_BSO_ may help maintain sexual desire, arousal, orgasm, lubrication, satisfaction, and pain at levels comparable to AMC. These findings are consistent with previous research using the FSFI (Islam et al., 2021); however, they contrast with others showing that RRBSO + ET_BSO_ had lower pleasure scores than AMC (Johansen et al., 2016; Madalinska et al., 2005). Again, these discrepancies may be due to different scales and some of these studies corrected for multiple comparisons (Madalinska et al., 2005) while others did not (Islam et al., 2021; Johansen et al., 2016).

#### Female Sexual Function Index and Age

To our knowledge, this is the first study to compare the sexual function of younger midlife women with early-life RRBSO + /–ET_BSO_ to older SM women with intact ovaries. Contrary to our hypotheses, we found no differences between RRBSO + /–ET_BSO_ and SM, suggesting that the sexual function of women with RRBSO, irrespective of ET_BSO_, is comparable to older SM women.

These findings contrast with previous research reporting poorer sexual function after RRBSO compared to SM (Benshushan et al., 2009). This difference may be due to that study not adjusting for multiple comparisons, or differences in sexual dysfunction scales. Our results do support prior studies showing similar sexual function between SM and women with early-life BSO for benign reasons using the FSFI (also no formal report of multiple comparison adjustment; Bıldırcın et al., 2020), as well as older women with a history of early-life BSO, irrespective of ET (Erekson et al., 2012) or HT (Kokcu et al., 2015). Observing this pattern within our cohort suggests that sexual function in women with early-life RRBSO + /–ET_BSO_ decreases rather rapidly. The equivalence of their sexual function with that of SM—who are on average 10 years older—suggests that these difficulties may persist across the lifespan unless successfully treated.

Notably, age had no effect on the FSFI_TOTAL_ nor on any of the FSFI domains, suggesting that age alone is not a significant contributor to sexual dysfunction. While decreased sexual function is often attributed to age, menopausal (or endocrine) status may additionally or uniquely shape sexual function (Avis et al., 2009; Dąbrowska-Galas et al., 2019; Dennerstein et al., 1994, 2001; Hayes & Dennerstein, 2005; Lindau et al., 2007).

#### Female Sexual Function Index and Mood

Our study supports the link between depressed mood and sexual dysfunction. We found no group differences in mood as measured by the CES-D; all group averages were in the mild depression range, aligning with prior studies of RRBSO and SM women (Gibson et al., 2012; Hickey et al., 2021b; Lin et al., 2020; Powell et al., 2020). However, our findings contrast previous reports of elevated depressive mood in RRBSO women compared to AMC (Hickey et al., 2016). The lack of group differences in our study may be due to a recruitment bias with people joining because they had mild mood issues.

While the CES-D has been inversely associated with the FSFI_TOTAL_ in young adult female medical students (Shindel et al., 2011) and female cancer survivors (Baser et al., 2012), we are the first to report this inverse association among *BRCA1/2* women with RRBSO. The inverse association between the CES-D and the FSFI arousal, lubrication, satisfaction, and pain domains mirrors previous research using the Hospital Anxiety and Depression Scale (Chan et al., 2019). Additionally, the lack of association between the CES-D and the FSFI desire and orgasm domains aligns with previous studies showing that orgasm and sexual desire in women of different menopausal statuses and ages (between ages 20 and 70) are unaffected by mood (Hayes et al., 2008; Jonusiene et al., 2013). However, it contrasts other studies showing that orgasm and desire decline as depressive symptoms increase (Jonusiene et al., 2013; Leeners et al., 2014; Llaneza et al., 2011; Pérez-Herrezuelo et al., 2020; Polland et al., 2019)—effects observed in RRBSO + HT_BSO_ (HT_BSO_ type unspecified; Chan et al., 2019). Discrepancies between studies may be due to the use of different depression and sexual function inventories as well as participant ages. Whether it is depression that increases sexual difficulties or sexual difficulties that lead to depression is something our study cannot determine.

#### Female Sexual Distress Scale-Revised

To our knowledge, this is the first study to compare sexual distress on the FSDS-R between RRBSO + /–ET_BSO_ and AMC and older SM while considering the role of depressed mood. The mean FSDS-R scores of RRBSO + /–ET_BSO_ and SM indicate clinically significant sexual distress (DeRogatis et al., 2008), which is in keeping with the literature (Dennerstein et al., 2006; Kalmbach et al., 2012; Leiblum et al., 2006; Tucker et al., 2016).

While we hypothesized that RRBSO would report higher sexual distress scores than RRBSO + ET_BSO_ and AMC, they only showed significantly higher scores than AMC. This finding suggests that ET_BSO_ may not successfully alleviate sexual distress for RRBSO women, aligning with previous reports that sexual distress does not differ between RRBSO taking and not taking ET_BSO_ (Tucker et al., 2016). Yet, supporting our hypothesis, we also found that RRBSO + ET_BSO_ did not differ significantly from AMC. Therefore, exogenous ET_BSO_ may help some women with RRBSO—but not all—achieve the lower levels of sexual distress observed in AMC. This finding is in contrast with prior studies showing that—irrespective of ET—sexual distress was higher in women with early-life BO compared to AMC (Dennerstein et al., 2006; HT; Shifren et al., 2008). Differences may be due to different scales, HT formulations, and confounding factors, such as reasons for surgery and the inclusion of women with concomitant hysterectomy.

RRBSO may have reported more sexual distress due to underestimating or feeling ill-informed about the sexual sequela following surgery, or because they experienced more sexual difficulties prior to RRBSO (Brotto et al., 2012). Notably, factors beyond hormones can also affect sexual distress. Among reproductive aged women with a history of infertility, dissatisfaction with marriage and painful intercourse predict sexual distress (range 15–49 years; Heshmatnia et al., 2025)—which affect women with RRBSO more so than AMC (Johansen et al., 2016; Michalowska, 2022). Thus, the variability in women’s responses to ET_BSO_ may make it difficult to reach consensus on its usefulness for all.

#### Female Sexual Distress Scale-Revised and Age

Supporting our hypothesis, we find higher sexual distress scores in younger midlife women with RRBSO compared to older SM; however, RRBSO + ET_BSO_ do not differ from SM. While prior studies showed higher sexual distress in women with early-life ovarian removal, irrespective of ET_BSO_, compared to SM women with (Dennerstein et al., 2006) or without intact ovaries (Tucker et al., 2016), our findings suggest that ET_BSO_ may confer some benefit in alleviating distress relative to SM for some women but not all.

Notably, age was not a significant covariate, indicating that age did not drive the observed differences. The reasons for their menopause, namely as a cancer prophylaxis in the case of RRBSO, may explain the disparity; women with RRBSO have been found to report higher sexual distress than women with BSO for benign reasons (Brotto et al., 2012).

#### Female Sexual Distress Scale-Revised and Mood

We found that a higher CES-D score was positively associated with the FSDS-R, indicating that elevated depressed mood parallels greater sexual distress. To our knowledge, these results represent the first direct demonstration of a positive association between the CES-D and FSDS-R among RRBSO women. This finding aligns with previous research in other populations showing that as depressive symptoms increase, so does sexual distress (Knoepp et al., 2010; Raggio et al., 2014) and extends prior findings that poorer emotional well-being (Short Form Health Survey) is associated with greater sexual distress in RRBSO women by demonstrating a similar association with the CES-D (Tucker et al., 2016).

### Summary of Quantitative Results

The FSFI_TOTAL_ and FSDS-R were significantly worse for RRBSO than for AMC. As the severity of depression symptoms increased, total sexual function, arousal, lubrication, and satisfaction decreased, and pain and sexual distress increased. Thus, the severity of sexual dysfunction is closely tied to mood. The present study also suggests that ET_BSO_ is beneficial for some women in ameliorating sexual problems but not all; this underscores the intermediate phenotype observed for ET_BSO_ with respect to sexual function, which is also the case for certain cognitive domains in our larger cohort. In women with RRBSO, ET_BSO_ ameliorates spatial working memory declines but not those in verbal episodic memory (Gervais et al., 2020). Thus, in both the sexual and some cognitive domains, ET_BSO_ is not a silver bullet.

### Qualitative Results

Women shared sexual challenges when interviewed without being explicitly asked about them, suggesting that sex is a cardinal part of the participants’ lives with a wide-reaching impact. Only one of the 14 interviewees did not spontaneously describe any sexual issues after RRBSO; the fact that the majority brought these issues to the interviewer’s attention in spite of not being asked about it speaks to the salience of sexual function for this population.

Some themes support our quantitative findings: “No appetite,” “It’s dry–it’s not like it was,” and Sweeping pain, highlighting the interpersonal and social contexts within which they experienced disrupted sexual function. Other themes, namely Sagging marriage bed, Sex and the self, and Women care about sex flesh out the psychosocial repercussions of the sexual problems.

While participants expressed missing sex and intimacy, they often shared concerns about their relationships, suggesting that RRBSO affects their marriage as well. Importantly, women with RRBSO who feel supported in their primary relationship report higher sexual satisfaction, even in the presence of poor physical function (Lorenz et al., 2014). Additionally, some women in our study described intentionally increasing sexual activity to reduce painful intercourse. Sexual and relationship satisfaction may play a protective role in sustaining sexual engagement after RRBSO, representing important targets for intervention (Leiblum et al., 2006; Lorenz et al., 2014). However, such relational concerns may also reflect a broader tendency for heterosexual women to focus primarily on their partner’s desire, pleasure, and satisfaction, and consider sexual interactions as an obligation they must fulfill (Herbenick et al., 2019; van Anders et al., 2022). Sex after RRBSO may bring this convention to the forefront, presenting even greater difficulties for women with RRBSO.

### Quantitative and Qualitative

The qualitative narratives described vaginal dryness, pain, and distress, regardless of ET_BSO_, mirroring our findings on the FSFI and the FSDS-R. Pain during intercourse and vaginal burning have been reported in other qualitative accounts of women with RRBSO (Hallowell et al., 2012; Meiser et al., 2000). Notably, of the eight participants who completed both components, the most discussed sexual experience among those meeting the cutoffs on the FSFI_TOTAL_ and FSDS-R was decreased sexual desire, suggesting it may be the most distressing change. In our study, women described this loss of desire as a sense of numbness, characterized by decreased motivation and sensation, regardless of ET_BSO_. Their experience paints a picture of probable HSDD; one marked by depressive mood, feelings of emptiness, and loss of intimacy that can lead to a crumbling of marital relationships.

### Qualitative Extensions of Quantitative Findings

We learn that even RRBSO + ET_BSO_ are concerned about their sex lives in which they also describe a marked loss of desire, sexual pain, and relational challenges. This may be due to the loss of endogenous E2, but it may also be that recurring memory of cancer risk, suggesting that cancer risk itself may contribute to sexual pain and accompanying distress. Notably, women with BSO as a cancer prophylaxis report disproportionately higher sexual distress than those with BSO for benign reasons (Brotto et al., 2012). Thus, when considering the diagnosis of HSDD in women with BSO it might be important to factor in the reason for BSO.

Further, a loss of sexual desire may extend beyond intercourse, but more broadly to a lack of physical and emotional intimacy that includes the simple act of touching. Only Camille, who was taking ET_BSO_ plus T, felt she had dealt successfully with low desire. Compared to ET_BSO_ only or placebo, T only and combined ET_BSO_–T was linked to greater desire, arousal, and sexual fantasies (Sherwin et al., 1985). In an open-label study, women with BSO taking combined ET_BSO_–T reported higher desire, arousal, and sexual fantasy scores than ET_BSO_ only or those untreated (Sherwin & Gelfand, 1987).

Subsequent randomized double-blind placebo-controlled trials in women with BSO and HSDD showed that transdermal T at 300 μg/day, with concomitant ET_BSO_, increased satisfying sexual activity, desire, orgasm, arousal, responsiveness, and self-image, and reduced sexual concerns and related distress, compared to placebo (oral ET_BSO_, Braunstein et al., 2005; oral or transdermal ET_BSO_, Buster et al., 2005; Simon et al., 2005; transdermal ET_BSO_, Davis et al., 2006). In SM and BSO women with HSDD not taking ET, 300 μg/day of transdermal T increased the frequency of satisfying sexual events and desire, while reducing distress (Davis et al., 2008). Further, intramuscular T (25 mg) improved sexual desire, arousal, and sexual activity (Huang et al., 2014).

Notably, an open-label extension of these trials (Buster et al., 2005; Simon et al., 2005) showed no increase in the rate of adverse events up to four years of treatment (Nachtigall et al., 2011). Although T remains unapproved for use in women by several national drug regulatory authorities, consensus statements and guidelines support its efficacy and safety when administered as a transdermal patch at doses approximating premenopausal physiologic concentrations, with or without ET, to treat HSDD in postmenopausal SM and BSO women (Davis et al., 2019; Parish et al., 2021).

To date, HSDD is the primary accepted indication for T with or without ET in postmenopausal (SM or BSO) women (Achilli et al., 2017; Davis et al., 2019; Islam et al., 2019; Parish et al., 2021; Simon & Ohleth, 2025). However, long-term safety data remain limited, and female-specific T treatment options remain an understudied and unmet clinical need (Davis et al., 2019; Islam et al., 2019; Parish et al., 2021; Simon & Ohleth, 2025).

Through participants’ accounts we also learned that their sex lives are dynamically experienced through their relationships with their husbands and socially situated bodies. Some women felt their partner’s support could falter with the emergence of sexual issues post-RRBSO, consistent with prior reports (Brotto et al., 2012; Hickey et al., 2021a). Others expressed feeling prematurely aged and pointed out how media and society link sex drive to energy, youth, and healthy relationships, aligning with other qualitative accounts of RRBSO women experiencing feelings of compromised femininity after surgery (Hallowell, 2000; Pernet et al., 1992). Further, apart from simple yes or no reports of occurrence, vaginal dryness was interwoven with distress about menopause and aging. Thus, the prevalent referral of BSO as “surgical menopause” is incorrect (Edwards et al., 2019; Rocca et al., 2021) and may engender distress and exacerbate a sense of aging. The experience of dryness thus extends beyond a loss of lubrication; it reinforces an aging sense of self as young and middle-aged women navigate what they feel to be accelerated aging.

The wide variability in our participants’ attempts to mitigate their failing sexual lives underscores a sense of agency and the dynamic interplay between the biological and the social factors that shape sexual pain (McCabe et al., 2010; Meana & Binik, 2022). Some relied, at least in part, on medication, and others sought to actively engage in sexual intercourse regularly. This variability further suggests that women are unsure of the best course of treatment and do not seek treatment from providers, which may be the result of barriers to accessing information or evidence-based interventions. Indeed, *BRCA1/2* women with RRBSO often underreport sexual and intimacy symptoms; barriers to disclosure include concerns of physicians feeling too embarrassed to discuss the topic and guilt in addressing sexual side effects in light of cancer risk reduction (Alexandre et al., 2017; Campfield Bonadies et al., 2011). The onus, consequently, seems to be on patients to help each other and report problematic symptoms even if they might be embarrassing (Hickey et al., 2021a). Notably, many healthcare providers believe that patients will raise the topic of sexual dysfunction and menopause if they are symptomatic (Ussher et al., 2013). Thus, sexuality often goes undiscussed with women.

Accordingly, as in other studies, women expressed a need for more information on sexual function post-RRBSO (Brotto, 2012; Campfield Bonadies et al., 2011), potential loss of libido (Hallowell, 2000; Hallowell et al., 2012), effects of HT (Meiser et al., 2000), menopause symptoms (Armon et al., 2023), and pain (Brotto, 2012). Like others, our participants reported being uninformed of the subsequent changes in their sex life, including the persistence and magnitude of loss of desire (Campfield Bonadies et al., 2011; Hallowell et al., 2012). Women are thus still not receiving the information they need; this lack of health-related communication and stigma surrounding female sexuality may have thwarted the process of informed consent.

### Strengths and Weaknesses

A major strength of this study is that using online quantitative questionnaires and in-person qualitative interviews afforded a comprehensive mixed-methods account of sexual changes following RRBSO, in some cases, in the same participant. By combining quantitative findings with the spontaneous comments on sexual function from qualitative interviews, this study provides insight into the meaning and repercussions of some of RRBSO’s sexual sequela. Together these approaches allow us to match the physiological expression with feelings, and interpersonal challenges of RRBSO, many of which may go undetected by quantitative questionnaires alone.

This study also had some limitations. First, we used self-administered questionnaires, which can introduce bias. In particular, the FSFI has a dysfunction bias (Meston et al., 2020; Meyer-Bahlburg & Dolezal, 2007). In addition, not all participants contributed to both the quantitative and qualitative components. While eight of the 14 qualitative participants also took the quantitative survey, to achieve adequate cohort size the quantitative survey was administered online and distributed to the in-person cohort; however, not all participants who were interviewed completed the quantitative portion. Finally, although the study was adequately powered to detect moderate to large effects, smaller group differences may have gone undetected, which should be considered when interpreting nonsignificant comparisons.

Moreover, discussion of sexual function in the qualitative interviews might not have been as far-reaching as it would have been had they been prompted to discuss it. We also lacked data on participants’ sexual function prior to RRBSO, preventing us from understanding how sexual function prior to RRBSO might affect the outcome of sexual function and distress.

Further, despite the quantitative component being widely available online, our cohort primarily comprised Canadian, Caucasian, and highly educated women. While our findings can only be applied to this group, it does add information to the Canadian context of having RRBSO. We also used the CES-D, which could be influenced by social desirability bias and is not a diagnostic tool for depression; instead, we know only about depressed mood within a week of testing (Radloff, 1977). In addition, because we did not quantify the dosage of ET_BSO_ or E2 levels, it is difficult for us to draw causal conclusions about how E2 does or does not affect sexual function; cohort-wide variations in dosage with few additions of T as well may have influenced the findings (Waaseth et al., 2008). However, since all but three participants were taking ET_BSO_ only we were able to assess the efficacy of ET_BSO_ alone to alleviate sexual dysfunction and related distress. Related to hormone efficacy, since only two participants in the quantitative study and one in the qualitative were taking concurrent T, we were unable to assess its effects on sexual dysfunction. Since RRBSO + ET_BSO_ participants were neither significantly different from AMC nor RRBSO, it would be worthwhile for future studies to determine whether the addition of T improves sexual function significantly.

### Conclusions

Quantitative and qualitative studies across women of all ages with RRBSO have long reported domain specific as well as overall poor sexual function and sexual distress post-RRBSO. In addition, depressed mood is associated with worse sexual function and distress. This study supports and extends these findings to a comparison to older women in SM with intact ovaries and provides a deeper understanding of the limits of one specific hormone treatment, ET_BSO_. Taken together, the interrelationships between depressed mood, age, sexual function and ET_BSO_ warrant further investigation, as does the need for additional intervention studies aimed at alleviating sexual dysfunction and related distress.

Perhaps most importantly, although sexual dysfunction has long been documented as a common sequela of RRBSO, our participants reported a lack of presurgical awareness of how RRBSO would affect their sexual and intimate life, which is surprising given previously published reports extending back to 1992 (Pernet et al., 1992). Since an earlier paper showed that women with preoperative knowledge of the sexual side effects following RRBSO had fewer sexual function problems and related distress, it would seem even more important for women to know about possible sexual corollaries of RRBSO (Brotto et al., 2012). Taken together, both our quantitative and qualitative findings underscore the urgency for physicians and healthcare personnel to provide full disclosure of possible sexual dysfunction for fully informed consent. Women value their sexual lives, and fully informed consent necessitates understanding the possible corollary effects of RRBSO on their intimate lives and sense of self.

## Electronic supplementary material

Below is the link to the electronic supplementary material.

Supplementary file1 (DOCX 31 KB)

## Appendix 1

### Interview Guide

Introductions, explanation of the rationale, and consent to record were obtained prior to starting the interview.

1. Could you start by telling me how you came to have an oophorectomy?

**Prompt**: As you remember the process, what stands out in your mind?

2. Prior to the surgery, what were you told about life after oophorectomy?

2a. What would you have liked to know?

3. What was it like entering menopause at [their age of BSO]?

**Probe:** If they mention feeling older, ask: What is your relationship with aging?

4. How has your own understanding of menopause changed with experiencing it?

**Prompt:** How has society and media influenced your understanding of menopause?

5. What has your body felt like after surgery? What changes have you experienced?

**Probe:** Follow-up with the changes they describe.

**Prompt:** Can you tell me about other changes you’ve experienced?

6. Have you noticed changes in your memory or concentration after surgery?

**Prompts:** Can you give me an example?

What does that feel like, [e.g., when you can’t remember names]

How do these changes make you feel?

Can you tell me about last time you noticed, this issue?

7. Are you taking hormone replacement therapy?

**If yes:** Can you talk about how that feels, and what your routine looks like?

8. Has your awareness of your body changed after surgery?

8a. Are you aware of the absence of the ovaries?

9. Has your perception of your body changed after surgery?

10. Has your sense of self changed since surgery?

11. What advice would you give to others who are facing such a decision?/What would you want others to know more generally?

**Closing:** Before we wrap-up, is there anything else with respect to your experience that you’d like to discuss that we haven’t touched on, or anything else you’d like to elaborate on.

## References

[CR1] Achilli, C., Pundir, J., Ramanathan, P., Sabatini, L., Hamoda, H., & Panay, N. (2017). Efficacy and safety of transdermal testosterone in postmenopausal women with hypoactive sexual desire disorder: A systematic review and meta-analysis. *Fertility and Sterility,**107*(2), 475-482.e15. 10.1016/j.fertnstert.2016.10.028 27916205

[CR2] Alarslan, D., Sarandol, A., Cengiz, C., & Develioglu, O. H. (2011). Androgens and sexual dysfunction in naturally and surgically menopausal women. *Journal of Obstetrics and Gynaecology Research,**37*(8), 1027–1034. 10.1111/j.1447-0756.2010.01479.x 21481089

[CR3] Alexandre, M., Black, J., Whicker, M., Minkin, M. J., & Ratner, E. (2017). The management of sexuality, intimacy, and menopause symptoms (SIMS) after prophylactic bilateral salpingo-oophorectomy: How to maintain sexual health in “Previvors.” *Maturitas,**105*, 46–51. 10.1016/j.maturitas.2017.06.003 28602465

[CR4] American Psychiatric Association. (2013). *Diagnostic and statistical manual of mental disorders* (5th ed.). 10.1176/appi.books.9780890425596

[CR5] Armon, S., Miron-Shatz, T., Mor, P., Tomer, A., Levy-Lahad, E., Michaelson-Cohen, R., & Srebnik, N. (2023). BRCA carriers after risk-reducing bilateral salpingo-oophorectomy: Menopausal hormone therapy knowledge gaps, and the impact of physicians’ recommendations. *Climacteric,**26*(2), 154–160. 10.1080/13697137.2023.2173567 36866779

[CR6] Atlantis, E., & Sullivan, T. (2012). Bidirectional association between depression and sexual dysfunction: A systematic review and meta-analysis. *Journal of Sexual Medicine,**9*(6), 1497–1507. 10.1111/j.1743-6109.2012.02709.x 22462756

[CR7] Avis, N. E., Brockwell, S., Randolph, J. F., Jr., Shen, S., Cain, V. S., Ory, M., & Greendale, G. A. (2009). Longitudinal changes in sexual functioning as women transition through menopause: Results from the Study of Women’s Health Across the Nation. *Menopause,**16*(3), 442–452. 10.1097/gme.0b013e3181948dd0 PMC2908487 19212271

[CR8] Baser, R. E., Li, Y., & Carter, J. (2012). Psychometric validation of the Female Sexual Function Index (FSFI) in cancer survivors. *Cancer,**118*(18), 4606–4618. 10.1002/cncr.26739 PMC13345471 22359250

[CR9] Basson, R., Leiblum, S., Brotto, L., DeRogatis, L., Fourcroy, J., Fugl-Meyer, K., Graziottin, A., Heiman, J. R., Laan, E., Meston, C., Schover, L., van Lankveld, J., & Schultz, W. W. (2003). Definitions of women’s sexual dysfunction reconsidered: Advocating expansion and revision. *Journal of Psychosomatic Obstetrics and Gynaecology,**24*(4), 221–229. 10.3109/01674820309074686 14702882

[CR10] Benshushan, A., Rojansky, N., Chaviv, M., Arbel-Alon, S., Benmeir, A., Imbar, T., & Brzezinski, A. (2009). Climacteric symptoms in women undergoing risk-reducing bilateral salpingo-oophorectomy. *Climacteric,**12*(5), 404–409. 10.1080/13697130902780846 19479488

[CR11] Bıldırcın, F. D., Özdeş, E. K., Karlı, P., Özdemir, A. Z., & Kökçü, A. (2020). Does type of menopause affect the sex lives of women? *Medical Science Monitor,**26*, Article e921811. 10.12659/MSM.921811 PMC6977620 31907344

[CR12] Bober, S. L., Recklitis, C. J., Bakan, J., Garber, J. E., & Patenaude, A. F. (2015). Addressing sexual dysfunction after risk‐reducing salpingo‐oophorectomy: Effects of a brief, psychosexual intervention. *Journal of Sexual Medicine,**12*(1), 189–197. 10.1111/jsm.12713 PMC4304978 25311333

[CR13] Bowen, G. A. (2008). “Naturalistic inquiry and the saturation concept: A research note.” *Qualitative Research,**8*(1), 137–152. 10.1177/1468794107085301

[CR14] Braun, V., & Clarke, V. (2006). Using thematic analysis in psychology. *Qualitative Research in Psychology,**3*(2), 77–101. 10.1191/1478088706qp063oa

[CR15] Braun, V., & Clarke, V. (2019). Reflecting on reflexive thematic analysis. *Qualitative Research in Sport, Exercise and Health,**11*(4), 589–597. 10.1080/2159676X.2019.1628806

[CR16] Braunstein, G. D., Sundwall, D. A., Katz, M., Shifren, J. L., Buster, J. E., Simon, J. A., Bachman, G., Aguirre, O. A., Lucas, J. D., Rodenberg, C., Buch, A., & Watts, N. B. (2005). Safety and efficacy of a testosterone patch for the treatment of hypoactive sexual desire disorder in surgically menopausal women: A randomized, placebo-controlled trial. *Archives of Internal Medicine,**165*(14), 1582–1589. 10.1001/archinte.165.14.1582 16043675

[CR17] Brotto, L. A. (2010). The DSM diagnostic criteria for hypoactive sexual desire disorder in women. *Archives of Sexual Behavior,**39*(2), 221–239. 10.1007/s10508-009-9543-1 19777334

[CR18] Brotto, L. A., Branco, N., Dunkley, C., McCullum, M., & McAlpine, J. N. (2012). Risk-reducing bilateral salpingo-oophorectomy and sexual health: A qualitative study. *Journal of Obstetrics and Gynaecology Canada,**34*(2), 172–178. 10.1016/S1701-2163(16)35160-X 22340066

[CR19] Buster, J. E., Kingsberg, S. A., Buch, A., Rodenberg, C. A., Wekselman, K., Buch, A., Rodenberg, C. A., Wekselman, K., & Casson, P. (2005). Testosterone patch for low sexual desire in surgically menopausal women: A randomized trial. *Obstetrics and Gynecology,**105*(5), 944–952. 10.1097/01.AOG.0000158103.27672.0d 15863529

[CR20] Campfield Bonadies, D., Moyer, A., & Matloff, E. T. (2011). What I wish I’d known before surgery: BRCA carriers’ perspectives after bilateral salpingo-oophorectomy. *Familial Cancer,**10*(1), 79–85. 10.1007/s10689-010-9384-z 20852945

[CR21] Carpenter, J. S., Reed, S. D., Guthrie, K. A., Larson, J. C., Newton, K. M., Lau, R. J., Learman, L. A., & Shifren, J. L. (2015). Using an FSDS-R item to screen for sexually related distress: A MsFLASH analysis. *Sexual Medicine,**3*(1), 7–13. 10.1002/sm2.53 PMC4380908 25844170

[CR22] Chan, J. L., Senapati, S., Johnson, L. N. C., DiGiovanni, L., Voong, C., Butts, S. F., & Domchek, S. M. (2019). Risk factors for sexual dysfunction in BRCA mutation carriers following risk-reducing salpingo-oophorectomy. *Menopause,**26*(2), 132–139. 10.1097/GME.0000000000001176 PMC6335191 30020253

[CR23] Dąbrowska-Galas, M., Dąbrowska, J., & Michalski, B. (2019). Sexual dysfunction in menopausal women. *Sexual Medicine,**7*(4), 472–479. 10.1016/j.esxm.2019.06.010 PMC6963159 31371259

[CR24] Davis, S. R., Baber, R., Panay, N., Bitzer, J., Perez, S. C., Islam, R. M., Kaunitz, A. M., Kingsberg, S. A., Lambrinoudaki, I., Liu, J., Parish, S. J., Pinkerton, J., Rymer, J., Simon, J. A., Vignozzi, L., & Wierman, M. E. (2019). Global consensus position statement on the use of testosterone therapy for women. *Journal of Clinical Endocrinology and Metabolism,**104*(10), 4660–4666. 10.1210/jc.2019-01603 PMC6821450 31498871

[CR25] Davis, S. R., van der Mooren, M. J., van Lunsen, R. H. W., Lopes, P., Ribot, J., Rees, M., Moufarege, A., Rodenberg, C., Buch, A., & Purdie, D. W. (2006). Efficacy and safety of a testosterone patch for the treatment of hypoactive sexual desire disorder in surgically menopausal women: A randomized, placebo-controlled trial. *Menopause,**13*(3), 387–396. 10.1097/01.gme.0000179049.08371.c7 16735935

[CR26] Davis, S. R., Moreau, M., Kroll, R., Bouchard, C., Panay, N., Gass, M., Braunstein, G. D., Hirschberg, A. L., Rodenberg, C., Pack, S., Koch, H., Moufarege, A., Studd, J., & APHRODITE Study Team. (2008). Testosterone for low libido in postmenopausal women not taking estrogen. *New England Journal of Medicine,**359*(19), 2005–2017. 10.1056/NEJMoa0707302 18987368

[CR27] Dennerstein, L., Burrows, G. D., Wood, C., & Hyman, G. (1980). Hormones and sexuality: Effect of estrogen and progestogen. *Obstetrics and Gynecology,**56*(3), 316–322. 7422170

[CR28] Dennerstein, L., Dudley, E., & Burger, H. (2001). Are changes in sexual functioning during midlife due to aging or menopause? *Fertility and Sterility,**76*(3), 456–460. 10.1016/S0015-0282(01)01978-1 11532464

[CR29] Dennerstein, L., Koochaki, P., Barton, I., & Graziottin, A. (2006). Hypoactive sexual desire disorder in menopausal women: A survey of Western European women. *Journal of Sexual Medicine,**3*(2), 212–222. 10.1111/j.1743-6109.2006.00215.x 16490014

[CR30] Dennerstein, L., Randolph, J., Taffe, J., Dudley, E., & Burger, H. (2002). Hormones, mood, sexuality, and the menopausal transition. *Fertility and Sterility,**77*(4), 42–48. 10.1016/S0015-0282(02)03001-7 12007901

[CR31] Dennerstein, L., Smith, A. M., Morse, C. A., & Burger, H. G. (1994). Sexuality and the menopause. *Journal of Psychosomatic Obstetrics & Gynecology,**15*(1), 59–66. 10.3109/01674829409025630 8038890

[CR32] DeRogatis, L., Clayton, A., Lewis-D’Agostino, D., Wunderlich, G., & Fu, Y. (2008). Validation of the Female Sexual Distress Scale-Revised for assessing distress in women with hypoactive sexual desire disorder. *Journal of Sexual Medicine,**5*(2), 357–364. 10.1111/j.1743-6109.2007.00672.x 18042215

[CR33] DeRogatis, L., Pyke, R., McCormack, J., Hunter, A., & Harding, G. (2011). Does the Female Sexual Distress Scale-Revised cover the feelings of women with HSDD? *Journal of Sexual Medicine,**8*(10), 2810–2815. 10.1111/j.1743-6109.2011.02385.x 21771282

[CR34] DeRogatis, L. R., Rosen, R., Leiblum, S., Burnett, A., & Heiman, J. (2002). The Female Sexual Distress Scale (FSDS): Initial validation of a standardized scale for assessment of sexually related personal distress in women. *Journal of Sex & Marital Therapy,**28*(4), 317–330. 10.1080/00926230290001448 12082670

[CR35] Downe-Wamboldt, B. (1992). Content analysis: Method, applications, and issues. *Health Care for Women International,**13*(3), 313–321. 10.1080/07399339209516006 1399871

[CR36] Edwards, H., Duchesne, A., Au, A. S., & Einstein, G. (2019). The many menopauses: Searching the cognitive research literature for menopause types. *Menopause,**26*(1), 45–65. 10.1097/GME.0000000000001171 PMC6314513 29994973

[CR37] Elit, L., Esplen, M. J., Butler, K., & Narod, S. (2001). Quality of life and psychosexual adjustment after prophylactic oophorectomy for a family history of ovarian cancer. *Familial Cancer,**1*, 149–156. 10.1023/A:1021119405814 14574171

[CR38] Erekson, E. A., Martin, D. K., Zhu, K., Ciarleglio, M. M., Patel, D. A., Guess, M. K., & Ratner, E. S. (2012). Sexual function in older women after oophorectomy. *Obstetrics and Gynecology,**120*(4), 833–842. 10.1097/AOG.0b013e31826af3d1 PMC3631541 22996101

[CR39] Finch, A., Metcalfe, K., Chiang, J., Elit, L., McLaughlin, J., Springate, C., Demsky, R., Murphy, J., Rosen, B., & Narod, S. (2011). The impact of prophylactic salpingo-oophorectomy on menopausal symptoms and sexual function in women who carry a BRCA mutation. *Gynecologic Oncology,**121*(1), 163–168. 10.1016/j.ygyno.2010.12.326 21216453

[CR40] Fogle, R. H., Stanczyk, F. Z., Zhang, X., & Paulson, R. J. (2007). Ovarian androgen production in postmenopausal women. *Journal of Clinical Endocrinology and Metabolism,**92*(8), 3040–3043. 10.1210/jc.2007-0581 17519304

[CR41] Gervais, N. J., Au, A., Almey, A., Duchesne, A., Gravelsins, L., Brown, A., Reuben, R., Baker-Sullivan, E., Schwartz, D. H., Evans, K., Bernardini, M. Q., Eisen, A., Meschino, W. S., Foulkes, W. D., Hampson, E., & Einstein, G. (2020). Cognitive markers of dementia risk in middle-aged women with bilateral salpingo-oophorectomy prior to menopause. *Neurobiology of Aging,**94*, 1–6. 10.1016/j.neurobiolaging.2020.04.019 32497876

[CR42] Gibson, C. J., Joffe, H., Bromberger, J. T., Thurston, R. C., Lewis, T. T., Khalil, N., & Matthews, K. A. (2012). Mood symptoms after natural menopause and hysterectomy with and without bilateral oophorectomy among women in midlife. *Obstetrics and Gynecology,**119*(5), 935–941. 10.1097/AOG.0b013e31824f9c14 PMC3339661 22525904

[CR43] Grady, M. P. (1998). *Qualitative and action research: A practitioner handbook*. Phi Delta Kappa Educational Foundation.

[CR44] Graziottin, A. (2010). Menopause and sexuality: Key issues in premature menopause and beyond. *Annals of the New York Academy of Sciences,**1205*(1), 254–261. 10.1111/j.1749-6632.2010.05680.x 20840281

[CR45] Graziottin, A., & Leiblum, S. R. (2005). Biological and psychosocial pathophysiology of female sexual dysfunction during the menopausal transition. *Journal of Sexual Medicine,**2*(s3), 133–145. 10.1111/j.1743-6109.2005.00129.x 16422790

[CR46] Hall, E., Finch, A., Jacobson, M., Rosen, B., Metcalfe, K., Sun, P., Narod, S. A., & Kotsopoulos, J. (2019). Effects of bilateral salpingo-oophorectomy on menopausal symptoms and sexual functioning among women with a BRCA1 or BRCA2 mutation. *Gynecologic Oncology,**152*(1), 145–150. 10.1016/j.ygyno.2018.10.040 30414741

[CR47] Hallowell, N. (2000). A qualitative study of the information needs of high‐risk women undergoing prophylactic oophorectomy. *Psycho-Oncology,**9*(6), 486–495. 10.1002/1099-1611(200011/12)9:6<486::aid-pon478>3.0.co;2-y 11180583

[CR48] Hallowell, N., Baylock, B., Heiniger, L., Butow, P. N., Patel, D., Meiser, B., Saunders, C., & Price, M. A. (2012). Looking different, feeling different: Women’s reactions to risk-reducing breast and ovarian surgery. *Familial Cancer,**11*(2), 215–224. 10.1007/s10689-011-9504-4 22198037

[CR49] Hayes, R., & Dennerstein, L. (2005). The impact of aging on sexual function and sexual dysfunction in women: A review of population-based studies. *Journal of Sexual Medicine,**2*(3), 317–330. 10.1111/j.1743-6109.2005.20356.x 16422862

[CR50] Hayes, R. D., Dennerstein, L., Bennett, C. M., Sidat, M., Gurrin, L. C., & Fairley, C. K. (2008). Original research-women’s sexual health: Risk factors for female sexual dysfunction in the general population: Exploring factors associated with low sexual function and sexual distress. *Journal of Sexual Medicine,**5*(7), 1681–1693. 10.1111/j.1743-6109.2008.00838.x 18410300

[CR51] Herbenick, D., Bartelt, E., Fu, T-C- (Jane)., Paul, B., Gradus, R., Bauer, J., & Jones, R. (2019). Feeling scared during sex: Findings from a U.S. probability sample of women and men ages 14 to 60. *Journal of Sex & Marital Therapy,**45*(5), 424–439. 10.1080/0092623X.2018.1549634 30946623

[CR52] Heshmatnia, F., Azizi, M., Milani, H., Nikbakht, R., Kheiri, M., Tolomehr, H., & Shahhosseini, Z. (2025). Prevalence and correlates of female sexual dysfunction and sexual distress in reproductive-aged women: A systematic review and meta-analysis. *BMC Women’s Health,**25*(1), 451. 10.1186/s12905-025-03960-4 PMC12481727 41024089

[CR53] Hickey, I., Jha, S., & Wyld, L. (2021a). The psychosexual effects of risk-reducing bilateral salpingo-oophorectomy in female BRCA1/2 mutation carriers: A systematic review of qualitative studies. *Gynecologic Oncology,**160*(3), 763–770. 10.1016/j.ygyno.2020.12.001 33317909

[CR54] Hickey, M., Moss, K. M., Brand, A., Wrede, C. D., Domchek, S. M., Meiser, B., Mishra, G. D., & Joffe, H. (2021b). What happens after menopause? (WHAM): A prospective controlled study of depression and anxiety up to 12 months after premenopausal risk-reducing bilateral salpingo-oophorectomy. *Gynecologic Oncology,**161*(2), 527–534. 10.1016/j.ygyno.2021.02.001 33583580

[CR55] Hickey, M., Schoenaker, D. A., Joffe, H., & Mishra, G. D. (2016). Depressive symptoms across the menopause transition: Findings from a large population-based cohort study. *Menopause,**23*(12), 1287–1293. 10.1097/GME.0000000000000712 27552471

[CR56] Huang, G., Basaria, S., Travison, T. G., Ho, M. H., Davda, M., Mazer, N. A., Miciek, R., Knapp, P. E., Zhang, A., Collins, L., Ursino, M., Appleman, E., Dzekov, C., Stroh, H., Ouellette, M., Rundell, T., Baby, M., Bhatia, N. N., Khorram, O., Friedman, T., … Bhasin, S. (2014). Testosterone dose-response relationships in hysterectomized women with or without oophorectomy: effects on sexual function, body composition, muscle performance and physical function in a randomized trial. *Menopause*, *21*(6), 612–623. 10.1097/GME.0000000000000093 PMC4701202 24281237

[CR57] Hutchinson, S., & Wilson, H. S. (1992). Validity threats in scheduled semistructured research interviews. *Nursing Research,**41*(Mar/Apr 92), 117–119. 1549516

[CR58] Islam, R. M., Bell, R. J., Green, S., Page, M. J., & Davis, S. R. (2019). Safety and efficacy of testosterone for women: A systematic review and meta-analysis of randomised controlled trial data. *The Lancet. Diabetes & Endocrinology,**7*(10), 754–766. 10.1016/S2213-8587(19)30189-5 31353194

[CR59] Islam, R. M., Davis, S. R., Bell, R. J., Tejada-Berges, T., Wrede, C. D., Domchek, S. M., Meiser, B., Kirk, J., Krejany, E. O., & Hickey, M. (2021). A prospective controlled study of sexual function and sexually related personal distress up to 12 months after premenopausal risk-reducing bilateral salpingo-oophorectomy. *Menopause,**28*(7), 748–755. 10.1097/GME.0000000000001766 33739311

[CR60] Jiang, L., Wang, Y., Zhang, Y., Li, R., Wu, H., Li, C., Wu, Y., & Tao, Q. (2019). The reliability and validity of the Center for Epidemiologic Studies Depression Scale (CES-D) for Chinese university students. *Frontiers in Psychiatry,**10*, 315–315. 10.3389/fpsyt.2019.00315 PMC6537885 31178764

[CR61] Johansen, N., Liavaag, A. H., Tanbo, T. G., Dahl, A. A., Pripp, A. H., & Michelsen, T. M. (2016). Sexual activity and functioning after risk-reducing salpingo-oophorectomy: Impact of hormone replacement therapy. *Gynecologic Oncology,**140*(1), 101–106. 10.1016/j.ygyno.2015.11.016 26597462

[CR62] Jonusiene, G., Zilaitiene, B., Adomaitiene, V., Aniuliene, R., & Bancroft, J. (2013). Sexual function, mood and menopause symptoms in Lithuanian postmenopausal women. *Climacteric,**16*(1), 185–193. 10.3109/13697137.2012.682746 22845491

[CR63] Judd, H. L., Judd, G. E., Lucas, W. E., & Yen, S. S. (1974). Endocrine function of the postmenopausal ovary: Concentration of androgens and estrogens in ovarian and peripheral vein blood. *Journal of Clinical Endocrinology and Metabolism,**39*(6), 1020–1024. 10.1210/jcem-39-6-1020 4430702

[CR64] Kalmbach, D. A., Ciesla, J. A., Janata, J. W., & Kingsberg, S. A. (2012). Specificity of anhedonic depression and anxious arousal with sexual problems among sexually healthy young adults. *Journal of Sexual Medicine,**9*(2), 505–513. 10.1111/j.1743-6109.2011.02533.x 22024317

[CR65] Karnieli-Miller, O., Strier, R., & Pessach, L. (2009). Power relations in qualitative research. *Qualitative Health Research,**19*(2), 279–289. 10.1177/1049732308329306 19150890

[CR66] Kaur, N., Malla, V. G., & Gupta, S. (2017). Serum estradiol level and postmenopausal symptoms in surgical and natural menopause. *International Journal of Reproduction, Contraception, Obstetrics and Gynecology,**6*(9), 3920-. 10.18203/2320-1770.ijrcog20174035

[CR67] Kingsberg, S. A., Larkin, L. C., & Liu, J. H. (2020). Clinical effects of early or surgical menopause. *Obstetrics and Gynecology,**135*(4), 853–868. 10.1097/AOG.0000000000003729 32168205

[CR68] Knight, R. G., Williams, S., McGee, R., & Olaman, S. (1997). Psychometric properties of the Centre for Epidemiologic Studies Depression Scale (CES-D) in a sample of women in middle life. *Behaviour Research and Therapy,**35*(4), 373–380. 10.1016/S0005-7967(96)00107-6 9134792

[CR69] Knoepp, L. R., Shippey, S. H., Chen, C. C., Cundiff, G. W., DeRogatis, L. R., & Handa, V. L. (2010). Sexual complaints, pelvic floor symptoms, and sexual distress in women over forty. *Journal of Sexual Medicine,**7*(11), 3675–3682. 10.1111/j.1743-6109.2010.01955.x PMC3163299 20704643

[CR70] Kokcu, A., Kurtoglu, E., Bildircin, D., Celik, H., Kaya, A., & Alper, T. (2015). Does surgical menopause affect sexual performance differently from natural menopause? *Journal of Sexual Medicine,**12*(6), 1407–1414. 10.1111/jsm.12891 25923516

[CR71] Krouwel, M., Jolly, K., & Greenfield, S. (2019). Comparing Skype (video calling) and in-person qualitative interview modes in a study of people with irritable bowel syndrome–An exploratory comparative analysis. *BMC Medical Research Methodology,**19*, Article 219. 10.1186/s12874-019-0867-9 PMC6883529 31783797

[CR72] Leeners, B., Hengartner, M. P., Rössler, W., Ajdacic-Gross, V., & Angst, J. (2014). The role of psychopathological and personality covariates in orgasmic difficulties: A prospective longitudinal evaluation in a cohort of women from age 30 to 50. *Journal of Sexual Medicine,**11*(12), 2928–2937. 10.1111/jsm.12709 25358802

[CR73] Leiblum, S. R., Koochaki, P. E., Rodenberg, C. A., Barton, I. P., & Rosen, R. C. (2006). Hypoactive sexual desire disorder in postmenopausal women: US results from the Womenʼs International Study of Health and Sexuality (WISHeS). *Menopause,**13*(1), 46–56. 10.1097/01.gme.0000172596.76272.06 16607098

[CR74] Lenth, R. V., & Piaskowski, J. (2025). *emmeans: Estimated marginal means, aka least‐squares means* (R package version 2.0.0) [Computer software]. https://rvlenth.github.io/emmeans/

[CR75] Lewinsohn, P. M., Seeley, J. R., Roberts, R. E., & Allen, N. B. (1997). Center for Epidemiologic Studies Depression Scale (CES-D) as a screening instrument for depression among community-residing older adults. *Psychology and Aging,**12*(2), 277–287. 10.1037/0882-7974.12.2.277 9189988

[CR76] Lin, K.Y.-H., Chou, C.-Y., Chang, C.Y.-Y., Lin, W.-C., & Wan, L. (2020). Association between oophorectomy and depression in patients with comorbidities: A nationwide cohort study in Taiwan. *Taiwanese Journal of Obstetrics & Gynecology,**59*(6), 899–905. 10.1016/j.tjog.2020.09.017 33218409

[CR77] Lindau, S. T., Schumm, L. P., Laumann, E. O., Levinson, W., O’Muircheartaigh, C. A., & Waite, L. J. (2007). A study of sexuality and health among older adults in the United States. *New England Journal of Medicine,**357*(8), 762–774. 10.1056/NEJMoa067423 PMC2426743 17715410

[CR78] Llaneza, P., Fernández-Iñarrea, J. M., Arnott, B., García-Portilla, M. P., Chedraui, P., & Pérez-López, F. R. (2011). Sexual function assessment in postmenopausal women with the 14-item Changes in Sexual Functioning Questionnaire. *Journal of Sexual Medicine,**8*(8), 2144–2151. 10.1111/j.1743-6109.2011.02309.x 21679299

[CR79] Long, C. Y., Liu, C. M., Hsu, S. C., Wu, C. H., Wang, C. L., & Tsai, E. M. (2006). A randomized comparative study of the effects of oral and topical estrogen therapy on the vaginal vascularization and sexual function in hysterectomized postmenopausal women. *Menopause,**13*(5), 737–743. 10.1097/01.gme.0000227401.98933.0b 16946685

[CR80] Lorenz, T., McGregor, B., & Swisher, E. (2014). Relationship satisfaction predicts sexual activity following risk-reducing salpingo-oophorectomy. *Journal of Psychosomatic Obstetrics and Gynaecology,**35*(2), 62–68. 10.3109/0167482X.2014.899577 PMC4117249 24693956

[CR81] Macbeth, D. (2001). On “reflexivity” in qualitative research: Two readings, and a third. *Qualitative Inquiry,**7*(1), 35–68. 10.1177/107780040100700103

[CR82] Madalinska, J. B., Hollenstein, J., Bleiker, E., van Beurden, M., Valdimarsdottir, H. B., Massuger, L. F., Gaarenstroom, K. N., Mourits, M. J., Verheijen, R. H., van Dorst, E. B., van der Putten, H., van der Velden, K., Boonstra, H., & Aaronson, N. K. (2005). Quality-of-life effects of prophylactic salpingo-oophorectomy versus gynecologic screening among women at increased risk of hereditary ovarian cancer. *Journal of Clinical Oncology,**23*(28), 6890–6898. 10.1200/JCO.2005.02.626 16129845

[CR83] Madalinska, J. B., van Beurden, M., Bleiker, E. M., Valdimarsdottir, H. B., Hollenstein, J., Massuger, L. F., Gaarenstroom, K. N., Mourits, M. J., Verheijen, R. H., van Dorst, E. B., van der Putten, H., van der Velden, K., Boonstra, H., & Aaronson, N. K. (2006). The impact of hormone replacement therapy on menopausal symptoms in younger high-risk women after prophylactic salpingo-oophorectomy. *Journal of Clinical Oncology,**24*(22), 3576–3582. 10.1200/JCO.2005.05.1896 16877724

[CR84] Marcinkowska, U. M., Shirazi, T., Mijas, M., & Roney, J. R. (2023). Hormonal underpinnings of the variation in sexual desire, arousal and activity throughout the menstrual cycle–A multifaceted approach. *Journal of Sex Research,**60*(9), 1297–1303. 10.1080/00224499.2022.2110558 36018001

[CR85] McCabe, M., Althof, S. E., Assalian, P., Chevret-Measson, M., Leiblum, S. R., Simonelli, C., & Wylie, K. (2010). Psychological and interpersonal dimensions of sexual function and dysfunction. *Journal of Sexual Medicine,**7*(1pt2), 327–336. 10.1111/j.1743-6109.2009.01618 20092442

[CR86] McCool, M. E., Zuelke, A., Theurich, M. A., Knuettel, H., Ricci, C., & Apfelbacher, C. (2016). Prevalence of female sexual dysfunction among premenopausal women: A systematic review and meta-analysis of observational studies. *Sexual Medicine Reviews,**4*(3), 197–212. 10.1016/j.sxmr.2016.03.002 27871953

[CR87] Meana, M., & Binik, Y. M. (2022). The biopsychosocial puzzle of painful sex. *Annual Review of Clinical Psychology,**18*(1), 471–495. 10.1146/annurev-clinpsy-072720-014549 35216521

[CR88] Meiser, B., Tiller, K., Gleeson, M. A., Andrews, L., Robertson, G., & Tucker, K. M. (2000). Psychological impact of prophylactic oophorectomy in women at increased risk for ovarian cancer. *Psycho-Oncology,**9*(6), 496–503. 10.1002/1099-1611(200011/12)9:6<496::AID-PON487>3.0.CO;2-Z 11180584

[CR89] Merleau-Ponty, M. (1962). *Phenomenology of perception* (D. A. Landes, trans.). Routledge.

[CR90] Meston, C. M., Freihart, B. K., Handy, A. B., Kilimnik, C. D., & Rosen, R. C. (2020). Scoring and interpretation of the FSFI: What can be learned from 20 years of use? *Journal of Sexual Medicine,**17*(1), 17–25. 10.1016/j.jsxm.2019.10.007 31735616

[CR91] Meyer-Bahlburg, H. F. L., & Dolezal, C. (2007). The Female Sexual Function Index: A methodological critique and suggestions for improvement. *Journal of Sex & Marital Therapy,**33*(3), 217–224. 10.1080/00926230701267852 17454519

[CR92] Michałowska, S. (2022). Difficult legacy in a close relationship. Sexual satisfaction, relationship satisfaction, and body image in patients with BRCA mutation after prophylactic mastectomy and/or adnexectomy. *Archives of Psychiatry and Psychotherapy,**24*(3), 7–19. 10.12740/APP/146346

[CR93] Modelska, K., Litwack, S., Ewing, S. K., & Yaffe, K. (2004). Endogenous estrogen levels affect sexual function in elderly post-menopausal women. *Maturitas,**49*(2), 124–133. 10.1016/j.maturitas.2003.12.007 15474756

[CR94] Nachtigall, L., Casson, P., Lucas, J., Schofield, V., Melson, C., & Simon, J. A. (2011). Safety and tolerability of testosterone patch therapy for up to 4 years in surgically menopausal women receiving oral or transdermal oestrogen. *Gynecological Endocrinology,**27*(1), 39–48. 10.3109/09513590.2010.487597 21142609

[CR95] Nathorst-Böös, J., Wiklund, I., Mattsson, L. -Å., Sandin, K., & von Schoultz, B. (1993). Is sexual life influenced by transdermal estrogen therapy?: A double blind placebo controlled study in postmenopausal women. *Acta Obstetricia et Gynecologica Scandinavica,**72*(8), 656–660. 10.3109/00016349309021160 8259754

[CR96] National Institute for Health and Care Excellence. (2019). *Familial breast cancer: Classification, care and managing breast cancer and related risks in people with a family history of breast cancer (NICE Clinical Guidelines No. 164)*. https://www.nice.org.uk/guidance/CG164 31940157

[CR97] Novick, G. (2008). Is there a bias against telephone interviews in qualitative research? *Research in Nursing & Health,**31*(4), 391–398. 10.1002/nur.20259 PMC3238794 18203128

[CR98] Parish, S. J., & Hahn, S. R. (2016). Hypoactive Sexual Desire Disorder: A review of epidemiology, biopsychology, diagnosis, and treatment. *Sexual Medicine Reviews,**4*(2), 103–120. 10.1016/j.sxmr.2015.11.009 27872021

[CR99] Parish, S. J., Simon, J. A., Davis, S. R., Giraldi, A., Goldstein, I., Goldstein, S. W., Kim, N. N., Kingsberg, S. A., Morgentaler, A., Nappi, R. E., Park, K., Stuenkel, C. A., Traish, A. M., & Vignozzi, L. (2021). International Society for the Study of Women’s Sexual Health clinical practice guideline for the use of systemic testosterone for hypoactive sexual desire disorder in women. *Journal of Women’s Health,**30*(4), 474–491. 10.1089/jwh.2021.29037 PMC8064950 33797277

[CR100] Pérez-Herrezuelo, I., Aibar-Almazán, A., Martínez-Amat, A., Fábrega-Cuadros, R., Díaz-Mohedo, E., Wangensteen, R., & Hita-Contreras, F. (2020). Female sexual function and its association with the severity of menopause-related symptoms. *International Journal of Environmental Research and Public Health,**17*(19). 10.3390/ijerph17197235 PMC7579461 33022931

[CR101] Pernet, A. L., Wardle, J., Bourne, T. H., Whitehead, M. I., Campbell, S., & Collins, W. P. (1992). A qualitative evaluation of the experience of surgery after false positive results in screening for familial ovarian cancer. *Psycho-Oncology,**1*(4), 217–233. 10.1002/pon.2960010405

[CR102] Philp, L., Alimena, S., Ferris, W., Saini, A., Bregar, A. J., del Carmen, M. G., Eisenhauer, E. L., Growdon, W. B., Goodman, A., Dorney, K., Mazina, V., & Sisodia, R. C. (2022). Patient reported outcomes after risk-reducing surgery in patients at increased risk of ovarian cancer. *Gynecologic Oncology,**164*(2), 421–427. 10.1016/j.ygyno.2021.12.017 34953629

[CR103] Polland, A. R., Davis, M., Zeymo, A., & Iglesia, C. B. (2019). Association between comorbidities and female sexual dysfunction: Findings from the third National Survey of Sexual Attitudes and Lifestyles (Natsal-3). *International Urogynecology Journal,**30*(3), 377–383. 10.1007/s00192-018-3739-7 30178126

[CR104] Powell, C. B., Alabaster, A., Le, A., Stoller, N., Armstrong, M. A., & Raine‐Bennett, T. (2020). Sexual function, menopausal symptoms, depression and cancer worry in women with BRCA mutations. *Psycho-Oncology,**29*(2), 331–338. 10.1002/pon.5253 31654466

[CR105] Radloff, L. S. (1977). The CES-D Scale: A self-report depression scale for research in the general population. *Applied Psychological Measurement,**1*(3), 385–401. 10.1177/014662167700100306

[CR106] Raggio, G. A., Butryn, M. L., Arigo, D., Mikorski, R., & Palmer, S. C. (2014). Prevalence and correlates of sexual morbidity in long-term breast cancer survivors. *Psychology & Health,**29*(6), 632–650. 10.1080/08870446.2013.879136 PMC5521019 24404999

[CR107] Roberts, R. E., & Vernon, S. W. (1983). The Center for Epidemiologic Studies Depression Scale: Its use in a community sample. *The American Journal of Psychiatry,**140*(1), 41–46. 10.1176/ajp.140.1.41 6847983

[CR108] Rocca, W. A., Grossardt, B. R., Geda, Y. E., Gostout, B. S., Bower, J. H., Maraganore, D. M., de Andra, M., & Melton, L. J., 3rd. (2008). Long-term risk of depressive and anxiety symptoms after early bilateral oophorectomy. *Menopause,**15*(6), 1050–1059. 10.1097/gme.0b013e318174f155 18724263

[CR109] Rocca, W. A., Grossardt, B. R., Geda, Y. E., Gostout, B. S., Bower, J. H., Maraganore, D. M., de Andra, M., & Melton, L. J., III. (2018). Long-term risk of depressive and anxiety symptoms after early bilateral oophorectomy. *Menopause,**25*(11), 1275–1285. 10.1097/GME.0000000000001229 30358723

[CR110] Rocca, W. A., Mielke, M. M., Gazzuola Rocca, L., & Stewart, E. A. (2021). Premature or early bilateral oophorectomy: A 2021 update. *Climacteric,**24*(5), 466–473. 10.1080/13697137.2021.1893686 PMC8532166 33719814

[CR111] Rosen, R., Brown, C., Heiman, J., Leiblum, S., Meston, C., Shabsigh, R., Ferguson, D., & D’Agostino, R., Jr. (2000). The Female Sexual Function Index (FSFI): A multidimensional self-report instrument for the assessment of female sexual function. *Journal of Sex & Marital Therapy,**26*(2), 191–208. 10.1080/009262300278597 10782451

[CR112] Santoro, N., Worsley, R., Miller, K. K., Parish, S. J., & Davis, S. R. (2016). Role of estrogens and estrogen-like compounds in female sexual function and dysfunction. *Journal of Sexual Medicine,**13*(3), 305–316. 10.1016/j.jsxm.2015.11.015 26944462

[CR113] Secoșan, C., Balint, O., Pirtea, L., Grigoraș, D., Bălulescu, L., & Ilina, R. (2019). Surgically induced menopause—A practical review of literature. *Medicina,**55*(8), Article 482. 10.3390/medicina55080482 PMC6722518 31416275

[CR114] Semmens, J. P., & Wagner, G. (1982). Estrogen deprivation and vaginal function in postmenopausal women. *Journal of the American Medical Association,**248*(4), 445–448. 10.1001/jama.1982.03330040033027 6283189

[CR115] Sheppard, L. C., & Raby, R. (2024). Off track or on point? Side comments in focus groups with teens. *Qualitative Research : QR,**24*(3), 628–646. 10.1177/14687941231176931 PMC11161323 38855362

[CR116] Sherwin, B. B., & Gelfand, M. M. (1987). The role of androgen in the maintenance of sexual functioning in oophorectomized women. *Psychosomatic Medicine,**49*(4), 397–409. 10.1097/00006842-198707000-00009 3615768

[CR117] Sherwin, B. B., Gelfand, M. M., & Brender, W. (1985). Androgen enhances sexual motivation in females: A prospective, crossover study of sex steroid administration in the surgical menopause. *Psychosomatic Medicine,**47*(4), 339–351. 10.1097/00006842-198507000-00004 4023162

[CR118] Shifren, J. L., Monz, B. U., Russo, P. A., Segreti, A., & Johannes, C. B. (2008). Sexual problems and distress in United States women. *Obstetrics & Gynecology,**112*(5), 970–978. 10.1097/AOG.0b013e3181898cdb 18978095

[CR119] Shindel, A. W., Eisenberg, M. L., Breyer, B. N., Sharlip, I. D., & Smith, J. F. (2011). Sexual function and depressive symptoms among female North American medical students. *Journal of Sexual Medicine,**8*(2), 391–399. 10.1111/j.1743-6109.2010.02085.x PMC3565606 21054793

[CR120] Simon, J. A. (2011). Identifying and treating sexual dysfunction in postmenopausal women: The role of estrogen. *Journal of Women’s Health,**20*(10), 1453–1465. 10.1089/jwh.2010.2151 21819250

[CR121] Simon, J., Braunstein, G., Nachtigall, L., Utian, W., Katz, M., Miller, S., Waldbaum, A., Bouchard, C., Derzko, C., Buch, A., Rodenberg, C., Lucas, J., & Davis, S. (2005). Testosterone patch increases sexual activity and desire in surgically menopausal women with hypoactive sexual desire disorder. *Journal of Clinical Endocrinology and Metabolism,**90*(9), 5226–5233. 10.1210/jc.2004-1747 16014407

[CR122] Simon, J. A., & Ohleth, K. (2025). Testosterone for treating female sexual dysfunction. *Clinical Obstetrics and Gynecology,**68*(1), 60–67. 10.1097/GRF.0000000000000916 39690483

[CR123] Siyam, T., Ross, S., Campbell, S., Eurich, D. T., & Yuksel, N. (2017). The effect of hormone therapy on quality of life and breast cancer risk after risk-reducing salpingo-oophorectomy: A systematic review. *BMC Women’s Health,**17*(1), 22–22. 10.1186/s12905-017-0370-6 PMC5359830 28320467

[CR124] Sorensen, J., Bautista, K. E., Lamvu, G., & Feranec, J. (2018). Evaluation and treatment of female sexual pain: A clinical review. *Curēus,**10*(3), e2379–e2379. 10.7759/cureus.2379 PMC5969816 29805948

[CR125] Stanisz, M., Panczyk, M., Kurzawa, R., & Grochans, E. (2019). The effect of prophylactic adnexectomy on the quality of life and psychosocial functioning of women with the BRCA1/BRCA2 mutations. *International Journal of Environmental Research and Public Health,**16*(24), Article 4995. 10.3390/ijerph16244995 PMC6950418 31818005

[CR126] The North American Menopause Society. (2013). Management of symptomatic vulvovaginal atrophy: 2013 Position Statement of The North American Menopause Society. *Menopause,**20*(9), 888–902. 10.1097/GME.0b013e3182a122c2 23985562

[CR127] The North American Menopause Society. (2022). The 2022 hormone therapy position statement of The North American Menopause Society. *Menopause,**29*(7), 767–794. 10.1097/GME.0000000000002028 35797481

[CR128] Tucker, P. E., Bulsara, M. K., Salfinger, S. G., Tan, J.J.-S., Green, H., & Cohen, P. A. (2016). The effects of pre-operative menopausal status and hormone replacement therapy (HRT) on sexuality and quality of life after risk-reducing salpingo-oophorectomy. *Maturitas,**85*, 42–48. 10.1016/j.maturitas.2015.12.004 26857878

[CR129] Tucker, P. E., & Cohen, P. A. (2017). Review article: Sexuality and risk-reducing salpingo-oophorectomy. *International Journal of Gynecological Cancer,**27*(4), 847–852. 10.1097/IGC.0000000000000943 28333842

[CR130] Ussher, J. M., Perz, J., Gilbert, E., Wong, W. K. T., Mason, C., Hobbs, K., & Kirsten, L. (2013). Talking about sex after cancer: A discourse analytic study of health care professional accounts of sexual communication with patients. *Psychology & Health,**28*(12), 1370–1390. 10.1080/08870446.2013.811242 23805956

[CR131] van Anders, S. M., Herbenick, D., Brotto, L. A., Harris, E. A., & Chadwick, S. B. (2022). The heteronormativity theory of low sexual desire in women partnered with men. *Archives of Sexual Behavior,**51*, 391–415. 10.1007/s10508-021-02100-x PMC8382213 34426898

[CR132] Vermeulen, R. F. M., van Beurden, M., Kieffer, J. M., Bleiker, E. M. A., Valdimarsdottir, H. B., Massuger, L. F. A. G., Mourits, M. J. E., Gaarenstroom, K. N., van Dorst, E. B. L., van der Putten, H. W. H. M., & Aaronson, N. K. (2017). Hormone replacement therapy after risk-reducing salpingo-oophorectomy minimises endocrine and sexual problems: A prospective study. *European Journal of Cancer,**84*, 159–167. 10.1016/j.ejca.2017.07.018 28818705

[CR133] von Hippel, C., Adhia, A., Rosenberg, S., Austin, S. B., Partridge, A., & Tamimi, R. (2019). Sexual function among women in midlife: Findings from the Nurses’ Health Study II. *Women’s Health Issues,**29*(4), 291–298. 10.1016/j.whi.2019.04.006 PMC6663609 31130435

[CR134] Waaseth, M., Bakken, K., Dumeaux, V., Olsen, K. S., Rylander, C., Figenschau, Y., & Lund, E. (2008). Hormone replacement therapy use and plasma levels of sex hormones in the Norwegian Women and Cancer Postgenome Cohort - A cross-sectional analysis. *BMC Women’s Health,**8*(1). 10.1186/1472-6874-8-1 PMC2254595 18194511

[CR135] Wojnar, D. M., & Swanson, K. M. (2007). Phenomenology: An exploration. *Journal of Holistic Nursing,**25*(3), 172–180. 10.1177/0898010106295172 17724386

