# Supplementary material for: “There’s No Magic Pill to Help Me, Right?”: Sex After Early Midlife Bilateral Salpingo-Oophorectomy
Source: Arch Sex Behav. 2026 Oct 15;55(7):3033–58. doi: 10.1007/s10508-026-03548-5 (PMC13748021; doi:10.1007/s10508-026-03548-5)
Supplement: Supplementary file 1 [file 10508_2026_3548_MOESM1_ESM.docx]

**Supplementary Material**

**Table S1**

*Unadjusted Pairwise Group Comparisons for FSFI Total Scores*

| Group 1 | Group 2 | *B* | SE | *t* | *p* |
| --- | --- | --- | --- | --- | --- |
| **RRBSO** | **AMC** | **-7.75** | **2.81** | **-2.76** | **.007** |
| RRBSO+ET_BSO_ | AMC | -3.21 | 2.40 | -1.33 | .185 |
| RRBSO | RRBSO+ET_BSO_ | -4.54 | 3.00 | -1.51 | .133 |
| **RRBSO** | **SM** | **-6.90** | **3.23** | **-2.14** | **.035** |
| RRBSO+ET_BSO_ | SM | -2.36 | 3.33 | -0.71 | .480 |
| AMC | SM | 0.84 | 3.07 | 0.28 | .784 |

Note. Unstandardized regression coefficients (*B*) from a multiple linear regression model predicting FSFI_TOTAL_ scores by group, controlling for age and CES-D scores, *F*(5, 92) = 4.12, *p* = .002, *R²* = .18, *R²* adjusted = .14. Age was a non-significant covariate (*p* = .431) but CES-D was significant (*p* = .005). Unadjusted *p*-values are reported; bolded values indicate pairwise comparisons significant at the unadjusted level (*p* < .05) prior to Holm–Bonferroni correction. After correction, RRBSO had significantly lower FSFI_TOTAL_ scores than AMC (adjusted *p* = .042), but all other group comparisons were nonsignificant (all adjusted p > .05).

**Table S2**

*Unadjusted Pairwise Group Comparisons for FSFI Desire Scores*

| Group 1 | Group 2 | *B* | SE | *t* | *p* |
| --- | --- | --- | --- | --- | --- |
| **RRBSO** | **AMC** | **-0.74** | **0.32** | **-2.33** | **.022** |
| RRBSO+ET_BSO_ | AMC | -0.28 | 0.27 | -1.06 | .290 |
| RRBSO | RRBSO+ET_BSO_ | -0.45 | 0.34 | -1.34 | .184 |
| RRBSO | SM | -0.67 | 0.36 | -1.84 | .069 |
| RRBSO+ET_BSO_ | SM | -0.21 | 0.37 | -0.57 | .568 |
| AMC | SM | -0.07 | 0.34 | -0.21 | .837 |

Note. Unstandardized regression coefficients (*B*) from a multiple linear regression model predicting FSFI desire scores by group, controlling for age and CES-D scores, *F*(5, 96) = 2.55, *p* = .033, *R²* = .12, *R²* adjusted = .07. Age (*p* = .131) and CES-D score (*p* = .200) were non-significant covariates. Unadjusted *p*-values are reported; bolded values indicate pairwise comparisons significant at the unadjusted level (*p* < .05) prior to Holm–Bonferroni correction. After correction, all group comparisons were nonsignificant (all adjusted p > .05).

**Table S3**

*Unadjusted Pairwise Group Comparisons for FSFI Arousal Scores*

| Group 1 | Group 2 | *B* | SE | *t* | *p* |
| --- | --- | --- | --- | --- | --- |
| **RRBSO** | **AMC** | **-1.21** | **0.53** | **-2.29** | **.024** |
| RRBSO+ET_BSO_ | AMC | -0.38 | 0.45 | -0.84 | .402 |
| RRBSO | RRBSO+ET_BSO_ | -0.84 | 0.57 | -1.47 | .144 |
| RRBSO | SM | -1.21 | 0.61 | -1.98 | .051 |
| RRBSO+ET_BSO_ | SM | -0.37 | 0.63 | -0.59 | .557 |
| SM | AMC | -0.01 | 0.58 | -0.01 | .991 |

Note. Unstandardized regression coefficients (*B*) from a multiple linear regression model predicting FSFI arousal scores by group, controlling for age and CES-D scores, *F*(5, 95) = 2.82, *p* = .020, *R²* = .13, *R²* adjusted = .08. Age was a non-significant covariate (*p* = .536) but CES-D was significant (*p* = .023). Unadjusted *p*-values are reported; bolded values indicate pairwise comparisons significant at the unadjusted level (*p* < .05) prior to Holm–Bonferroni correction. After correction, all group comparisons were nonsignificant (all adjusted p > .05).

**Table S4**

*Unadjusted Pairwise Group Comparisons for FSFI Lubrication Scores*

| Group 1 | Group 2 | *B* | SE | *t* | *p* |
| --- | --- | --- | --- | --- | --- |
| **RRBSO** | **AMC** | **-1.75** | **0.66** | **-2.67** | **.009** |
| RRBSO+ET_BSO_ | AMC | -0.70 | 0.55 | -1.25 | .213 |
| RRBSO | RRBSO+ET_BSO_ | -1.06 | 0.70 | -1.50 | .137 |
| RRBSO | SM | -1.30 | 0.76 | -1.71 | .091 |
| RRBSO+ET_BSO_ | SM | -0.24 | 0.78 | -0.31 | .761 |
| SM | AMC | -0.46 | 0.72 | -0.64 | .527 |

Note. Unstandardized regression coefficients (*B*) from a multiple linear regression model predicting FSFI lubrication scores by group, controlling for age and CES-D scores, *F*(5, 95) = 3.26, *p* = .009, *R²* = .15, *R²* adjusted = .10. Age was a non-significant covariate (*p* = .940), but CES-D was significant (*p* = .016). Unadjusted *p*-values are reported; bolded values indicate pairwise comparisons significant at the unadjusted level (*p* < .05) prior to Holm–Bonferroni correction. After correction, all group comparisons were nonsignificant (all adjusted p > .05).

**Table S5**

*Unadjusted Pairwise Group Comparisons for FSFI Satisfaction Scores*

| Group 1 | Group 2 | *B* | SE | *t* | *p* |
| --- | --- | --- | --- | --- | --- |
| **RRBSO** | **AMC** | **-1.15** | **0.44** | **-2.64** | **.010** |
| **RRBSO+ET_BSO_** | **AMC** | **-0.80** | **0.37** | **-2.14** | **.035** |
| RRBSO | RRBSO+ET_BSO_ | -0.36 | 0.47 | -0.77 | .446 |
| RRBSO | SM | -0.81 | 0.50 | -1.63 | .107 |
| RRBSO+ET_BSO_ | SM | -0.46 | 0.52 | -0.89 | .377 |
| SM | AMC | -0.34 | 0.48 | -0.71 | .479 |

Note. Unstandardized regression coefficients (*B*) from a multiple linear regression model predicting FSFI satisfaction scores by group, controlling for age and CES-D scores, *F*(5, 92) = 5.93, *p* < .001, *R²* = .24, *R²* adjusted = .20. Age was not a significant covariate (*p* = .128), but CES-D was (*p* < .001). Unadjusted *p*-values are reported; bolded values indicate pairwise comparisons significant at the unadjusted level (*p* < .05) prior to Holm–Bonferroni correction. After correction, all group comparisons were nonsignificant (all adjusted p > .05).

**Table S6**

*Unadjusted Pairwise Group Comparisons for FSFI Pain Scores*

| Group 1 | Group 2 | *B* | SE | *t* | *p* |
| --- | --- | --- | --- | --- | --- |
| **RRBSO** | **AMC** | **-1.80** | **0.77** | **-2.35** | **.021** |
| **RRBSO+ET_BSO_** | **AMC** | **-0.95** | **0.65** | **-2.35** | **.021** |
| RRBSO | RRBSO+ET_BSO_ | -0.84 | 0.82 | -1.03 | .307 |
| **RRBSO** | **SM** | **-1.94** | **0.88** | **-2.20** | **.031** |
| RRBSO+ET_BSO_ | SM | -1.09 | 0.91 | -1.20 | .232 |
| SM | AMC | 0.14 | 0.84 | 0.17 | .869 |

Note. Unstandardized regression coefficients (*B*) from a multiple linear regression model predicting FSFI pain scores by group, controlling for age and CES-D scores, F(5, 94) = 3.47, p = .006, R² = .16, R² adjusted = .11. Age was a non-significant covariate (*p* = .203), but CES-D was (*p* = .014). Unadjusted *p*-values are reported; bolded values indicate pairwise comparisons significant at the unadjusted level (*p* < .05) prior to Holm–Bonferroni correction. After correction, all group comparisons were nonsignificant (all adjusted p > .05).

**Table S7**

*Unadjusted Pairwise Group Comparisons for FSDS-R Scores*

| Group 1 | Group 2 | *B* | SE | *t* | *p* |
| --- | --- | --- | --- | --- | --- |
| **RRBSO** | **AMC** | **12.35** | **2.60** | **4.59** | **< .001** |
| **RRBSO+ET_BSO_** | **AMC** | **5.82** | **2.27** | **2.57** | **.012** |
| **RRBSO** | **RRBSO+ET_BSO_** | **6.52** | **2.90** | **2.25** | **.027** |
| **RRBSO** | **SM** | **11.22** | **3.09** | **3.64** | **< .001** |
| RRBSO+ET_BSO_ | SM | 4.70 | 3.17 | 1.48 | .141 |
| SM | AMC | 1.12 | 2.91 | 0.39 | .701 |

Note. Unstandardized regression coefficients (*B*) from a multiple linear regression model predicting FSDS-R scores, controlling for age and CES-D scores, *F*(5, 97) = 11.47, *p* < .001, *R²* = .37, *R²* adjusted = .34. Age was a non-significant covariate (*p* = .378), but CES-D was significant (*p* < .001). Unadjusted *p*-values are reported; bolded values indicate pairwise comparisons significant at the unadjusted level (*p* < .05) prior to Holm–Bonferroni correction. After correction, RRBSO had significantly higher FSDS-R scores than AMC (adjusted *p* < .001) and SM (adjusted *p* = .002). All other group comparisons were nonsignificant (all adjusted p > .05).
